# Modifiable predictors of suicidal ideation during psychotherapy for late-life major depression. A machine learning approach

**DOI:** 10.1038/s41398-021-01656-5

**Published:** 2021-10-18

**Authors:** George S. Alexopoulos, Patrick J. Raue, Samprit Banerjee, Elizabeth Mauer, Patricia Marino, Mohamed Soliman, Dora Kanellopoulos, Nili Solomonov, Adenike Adeagbo, Jo Anne Sirey, Thomas D. Hull, Dimitris N. Kiosses, Patricia A. Areán

**Affiliations:** 1 Weill Cornell Institute of Geriatric Psychiatry, Weill Cornell Medicine, White Plains, NY USA; 2 Department of Psychiatry and Behavioral Sciences, University of Washington School of Medicine, Seattle, WA USA; 3 Talkspace, New York, NY USA; 4 Teachers College, Columbia University, New York, NY USA

**Keywords:** Depression, Human behaviour

## Abstract

This study aimed to identify subgroups of depressed older adults with distinct trajectories of suicidal ideation during brief psychotherapy and to detect modifiable predictors of membership to the trajectories of suicidal ideation. Latent growth mixed models were used to identify trajectories of the presence of suicidal ideation in participants to a randomized controlled trial comparing Problem Solving Therapy with “Engage” therapy in older adults with major depression over 9 weeks. Predictors of membership to trajectories of suicidal ideation were identified by the convergence of four machine learning models, i.e., least absolute shrinkage and selection operator logistic regression, random forest, gradient boosting machine, and classification tree. The course of suicidal ideation was best captured by two trajectories, a favorable and an unfavorable trajectory comprising 173 and 76 participants respectively. Members of the favorable trajectory had no suicidal ideation by week 8. In contrast, members of the unfavorable trajectory had a 60% probability of suicidal ideation by treatment end. Convergent findings of the four machine learning models identified hopelessness, neuroticism, and low general self-efficacy as the strongest predictors of membership to the unfavorable trajectory of suicidal ideation during psychotherapy. Assessment of suicide risk should include hopelessness, neuroticism, and general self-efficacy as they are predictors of an unfavorable course of suicidal ideation in depressed older adults receiving psychotherapy. Psychotherapeutic interventions exist for hopelessness, emotional reactivity related to neuroticism, and low self-efficacy, and if used during therapy, may improve the course of suicidal ideation.

## Introduction

Despite the recent increase of suicide in younger populations, older adults continue to have a suicide rate higher that of the general population [1]. In 2018, the annual suicide rate of persons aged 65 years and older was 39.9 per 100,000, compared to 14.1 per 100,000 in the general population.

Suicidal ideation and depression are risk factors for late-life suicide and targets for intervention. Genetic factors account for some part of the familial transmission of suicidal ideation, with estimates of heritability of 36% [2]. Suicidal ideation is co-transmitted with mood disorders. Depression is the most common psychiatric diagnosis in elderly suicide victims [3]. Most elderly victims of suicide have a mild to moderately severe depression, no previous depressive episodes, and no comorbid substance abuse or personality disorders [3]. These characteristics predict a favorable response to psychotherapy.

Efficacious psychotherapies for late-life major depression exist. Among them, problem-solving therapy (PST) has been shown to decrease depression more than supportive therapy [4]. In a randomized controlled trial, PST yielded one additional remission over supportive therapy for every 5.6 elderly patients with major depression and executive dysfunction and reduced suicidal ideation [5]. “Engage” is a novel, streamlined psychotherapy. It theorizes that dysfunction of reward networks is central to late-life depression, uses “reward exposure” as its main intervention, and assumes that repeated reward exposure can recondition the reward system and improve depression [6]. Negativity bias, apathy, and inadequate emotion regulation, reflecting dysfunction of the negative valence systems, the reward and salience networks, and the cognitive control network, respectively, are targeted when they serve as barriers to reward exposure. An effectiveness study found “Engage” to be noninferior to PST in reducing depression and in leading to response and remission of late-life depression [7].

This study reports on data of the randomized controlled trial that compared “Engage” with PST in late-life major depression [7] and had two aims. First, it sought to identify subgroups of depressed older adults with distinct trajectories of suicidal ideation during treatment. Second, it attempted to identify baseline predictors of the course of suicidal ideation. It used, to this end, four machine learning models because of their sensitivity and replicability of findings and their ability to detect complex (nonlinear) multidimensional interactions among predictors.

## Methods

This two-center, randomized clinical trial compared the efficacy of “Engage” to PST participants, recruited by Weill–Cornell Medicine and by the University of Washington (UW) between 2014 and 2019. The study was approved by the institutional review boards of both universities.

### Participants

Inclusion criteria were: 1) Age ≥60 years; 2) unipolar, nonpsychotic major depression (by SCID, DSM-IV) [8]; 3) Montgomery Åsberg Depression Rating Scale (MADRS) ≥20 [9]; 4) Mini Mental State Examination (MMSE) ≥24 [10]; 5) off antidepressants or on a stable dose of an antidepressant for 12 weeks and no plan to change the dose in the next 10 weeks; and 6) capacity to consent.

Exclusion Criteria were: 1) Intent or plan to attempt suicide imminently; 2) Psychiatric diagnoses other than unipolar, nonpsychotic major depression or generalized anxiety disorder; 3) Psychotropic drugs or cholinesterase inhibitors other than a stable course of antidepressants and mild doses of benzodiazepines, i.e., lorazepam up to 1.5 mg total daily dose or the equivalent of other benzodiazepines.

### Assessment

Raters were unaware of treatment condition and hypotheses. The primary outcome was suicidal ideation, assessed with the Hamilton Depression Rating Scale, Suicide Item (Rates 0 to 3) at baseline, and at approximately 2, 4, 6, 8, and 9 weeks of treatment. Potential predictors of suicidal ideation assessed at baseline were: Demographics, treatment assignment (“Engage” vs. PST), age of onset, length of current episode (months), number of previous episodes, severity of depression (MADRS minus the suicide item), disability [12-item World Health Organization Disability Assessment Schedule II (WHODAS-II) [11]; cognitive impairment [Mini-Mental Status Exam (MMSE); executive functioning [Initiation Perseveration Domain of Mattis Dementia Rating Scale (DRS) [12] and digit symbol substitution]; neuroticism (NEO Neuroticism subscale) [13]; apathy (Apathy Evaluation Scale) [14]; hopelessness [Beck Hopelessness Scale (BHS)] [15]; activation [subscale of the Behavioral Activation for Depression Scale (BADS) [16]]; avoidance/rumination (subscale of BADS); work/school impairment avoidance/rumination (subscale of BADS); social impairment (subscale of BADS); anhedonia (Snaith Hamilton Pleasure Scale) [17]; rumination response style scale [18]; and digit span [19].

### Treatments

Participants were randomized to either “Engage” or PST at each site using 1:1 randomly selected block sizes. A similar process was used to randomize therapists to Engage or PST. Therapists were aware of participants’ randomization but not of hypotheses. Participants received 9 weekly sessions of either treatment by 35 trained community social workers. Therapists had demonstrated fidelity and adherence to both the treatment manuals [7].

#### “Engage”

During Engage, patients and therapists develop a list of meaningful, rewarding activities, select two to three activities and make action plans for pursuing these activities between sessions. When patients do not engage or benefit from “reward exposure” during the first three sessions, therapists identify the primary barrier to reward exposure (negativity bias, apathy, or inadequate emotion regulation) and address it with defined strategies (stepped treatment). A second assessment of barriers to reward exposure occurs between sessions 3–6, and strategies targeting an emerging barrier are introduced when needed (Manual in Supplement).

#### Problem-solving therapy (PST)

PST trains participants in a 7-step problem-solving model, teaching them to set goals, develop ways to reach them, formulate action plans and assess their progress toward goals [20]. The last two sessions also focus on relapse prevention planning (PST Manual in Supplement).

### Data analysis

#### Trajectories of suicidal ideation during treatment

First, logistic mixed-effects models compared the probability of suicidal ideation between Engage-treated and PST-treated participants over the course of treatment. The mixed model used any suicidal ideation (HAM-D suicidal ideation item scores: 1, 2, or 3) as the dependent variable along with fixed effects of site, time, and treatment *x* time interaction and a subject-specific random intercept. The model was further adjusted for any observed imbalance of baseline covariates (i.e., hopelessness, neuroticism, and general self-efficacy) between the two treatment groups (*p* < = 0.05) that may influence treatment response.

Latent growth (logistic) mixed models (LGMM) were, then, used to discover latent subgroups with distinct trajectories of the probability of any suicidal ideation from baseline to treatment end after combining the two treatment groups. Four LGMM models were constructed with *K* = 2–5 trajectories, and for each of them, considered constant, linear, and quadratic time trend parameters. The best-fitting model was identified by the lowest Bayesian Information Criterion (BIC).

#### Baseline predictors of membership to suicidal ideation trajectories

Missing values of the baseline variables were imputed using the proximity measures of a random forest [21]. Initially, we compared baseline characteristics of the LGMM-identified latent trajectories of suicidal ideation using chi-square or independent two-sample *t* tests as appropriate. Then, we used four, complementary to each other, machine learning methods to identify baseline predictors of membership to trajectories of suicidal ideation, i.e., least absolute shrinkage and selection operator (LASSO) logistic regression, a random forest (RF) [22], a gradient boosting machine (GBM), and a classification tree [22, 23].

We used LASSO to model additive effects of predictors and classification tree to model complex interactions among predictors. We applied RF and GBM to improve the performance of classification trees by averaging over multiple classification trees. Specifically, LASSO is a penalized logistic regression model that selects important predictors by shrinking the coefficients of less important predictors to zero. Classification tree machine learning creates rules for prediction based on binary splits of predictors. The combination of classification rules defines the classification tree. RF and GBM assess prediction accuracy by considering an ensemble of trees. Specifically, RF averages multiple classification trees fit on multiple bootstrapped samples of data and randomly selects predictors for each split in a tree. GBM ensembles multiple trees by progressively fitting shallow trees or weak learners to the data and transforming the data in each step. Predictors are ranked on their relative “importance” by quantifying the improvement in prediction error by each predictor.

Prediction accuracy was operationalized using the area under the receiver operating characteristics curve (AU-ROC) and was estimated by the fivefold cross-validation (CV). The variability of the estimated AU-ROC is reported by computing the 95% confidence intervals of the AUC [24]. Tuning parameters for each of the four machine learning method were identified by embedding another 5-fold CV within the outer CV. The optimal tuning parameters for each method were chosen within each fold of the outer CV. The tuning parameters for the four machine learning methods were the shrinkage parameter for LASSO, the number of trees and the number of randomly chosen predictors for a candidate split in each tree for RF, tree depth, learning rate, and the minimum number of observations in a node for a split to happen for GBM, and the cost complexity and tree depth parameter for the classification tree.

## Results

The participants were 249 older adults (≥60 years) with major depression. They were randomly assigned to “Engage” or PST. The CONSORT chart and characteristics of participants were reported elsewhere [7] and appear in the Supplement (eFig. 1).

### Decline of suicidal ideation during “engage” and PST

Mixed-effects models showed that participants treated with Engage had a comparable decline in suicidal ideation (suicide item of HAM-D) to that of participants treated with PST chi square = 0.541, df = 1, 239, *p* = 0.462 for the treatment *x* time interaction). A Figure of the trajectories of suicidal ideation can be found in the Supplement (eFig. 2).

### Trajectories of response to psychotherapy

**Fig. 1:**
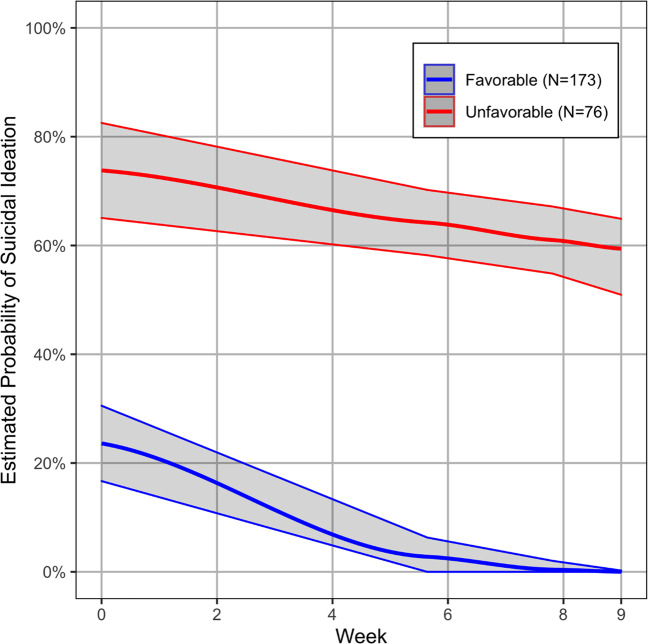
Latent growth mixture model (LGMM) estimated growth curves of the presence of suicidal ideation in 249 older adults with major depression randomly assigned to “engage” or problem-solving therapy. The figure presents two LGMM trajectories with 95% CI of the probability of the presence of suicidal ideation over 9 weeks. Red color represents an unfavorable (31% of participants) and blue color represents a favorable trajectory of suicidal ideation (69% of participants). The presence of suicidal ideation during the course of the 9-week treatment trial is defined as a score of 1, 2, or 3 in the Suicide item of the 24-item Hamilton Depression Rating Scale.

LGMM showed that the presence of suicidal ideation during treatment was best captured by two trajectories, a favorable and an unfavorable trajectory comprising 173 and 76 depressed participants respectively (Fig. 1). The likelihood of the presence of suicidal ideation was lower at baseline in members of the favorable compared to the unfavorable trajectory; the predicted probability of any suicidal ideation at the initial interview was 24% in the favorable trajectory, while the likelihood of any suicidal ideation in the unfavorable trajectory was 74%. Members of the favorable trajectory had no suicidal ideation by week 8. In contrast, participants of the unfavorable trajectory had at least a 60% probability of suicidal ideation by treatment end. The average posterior probability of membership to the favorable trajectory was 97% and that of membership to the unfavorable trajectory was 95%.

LGMM showed that symptoms of depression other than suicidal ideation (HAM-D minus the suicide item) also followed two trajectories (Supplemental Material, eFig. 3). Of those with a favorable trajectory of suicidal ideation, 32% had an unfavorable depression severity trajectory. Among those with an unfavorable suicidal ideation trajectory, 25% had a favorable depression severity trajectory.

### Predictors of the suicidal ideation trajectories: machine learning models

At baseline, participants with a favorable suicidal ideation trajectory had lower scores on hopelessness, neuroticism, the severity of depression, and higher scores of general self-efficacy, and behavioral activation (Table 1). However, demographic variables, site, age of depression onset, duration of current episode, anhedonia, rumination, apathy, history of antidepressant drug use, disability, cognitive scores, or treatment condition (“Engage vs. PST) were not significantly different across the two LGMM trajectories.

**Table 1:** Baseline clinical and demographic characteristics of members to two suicidal ideation trajectories identified by Latent Growth Mixture Model (LGMM).

	LGMM Trajectory of Suicidal Ideation		t/Chi-sq	*p* value^b^
	Favorable, *N* = 173^a^	Unfavorable, *N* = 76^a^		
**Site**			1.039	>0.999
Cornell	91 (53%)	46 (61%)		
UW	82 (47%)	30 (39%)		
**Treatment**			0.343	>0.999
Engage	87 (50%)	42 (55%)		
PST	86 (50%)	34 (45%)		
**Age (years)**	70 (7)	70 (8)	−0.099	>0.999
**Sex**			0.09	>0.999
Male	57 (33%)	23 (30%)		
Female	115 (67%)	53 (70%)		
**Ethnicity**			0	>0.999
Not Hispanic	161 (95%)	73 (96%)		
Hispanic	8 (4.7%)	3 (3.9%)		
**Race**			1.46	>0.999
white	153 (88%)	68 (89%)		
black	10 (5.8%)	2 (2.6%)		
other	10 (5.8%)	6 (7.9%)		
**Marital Status**			0.439	>0.999
unmarried	114 (67%)	46 (61%)		
married	57 (33%)	29 (39%)		
**Education (years)**	16 (3)	16 (2)	0.54	>0.999
**Age of Onset**	46 (24)	42 (24)	1.277	>0.999
**Length of Episode**	49 (109)	60 (135)	−0.603	>0.999
**MADRS**^**1**^	24.8 (4.0)	26.9 (4.6)	−3.534	0.017
**Hx Antidepressant Use**	111 (66%)	52 (68%)	0.046	>0.999
**Number of Sessions**	7.99 (2.29)	7.89 (2.35)	0.31	>0.999
**BADS**^**2**^**, Activation**	18 (8)	14 (7)	3.343	0.03
**BADSAvoidance/Rumination**	21 (8)	24 (8)	−2.63	0.273
**BADS, Work Impairment**	13 (7)	16 (6)	−2.748	0.195
**BADS, Social Impairment**	10 (7)	12 (6)	−2.209	0.829
**BHS**^**3**^	17.0 (4.7)	20.7 (3.9)	−6.338	<0.001
**AES**^**4**^	37 (8)	37 (8)	−0.453	>0.999
**NEO Neuroticism**	16.8 (4.8)	20.4 (4.1)	−5.935	<0.001
**Rumination**^**5**^	51 (9)	55 (9)	−2.624	0.279
**Pleasure Scale**^**6**^	2.43 (2.77)	2.94 (2.55)	−1.384	>0.999
**WHODAS**^**7**^	27 (8)	31 (8)	−3.098	0.068
**General Self-Efficacy**^**8**^	28.6 (5.1)	25.7 (5.3)	3.929	0.004
**MMSE**^**9**^	28.70 (1.27)	28.71 (1.35)	−0.038	>0.999
**DSS**^**10**^	42 (10)	42 (9)	−0.141	>0.999
**DRS-IP**^**11**^	35.0 (3.3)	35.4 (2.7)	−0.945	>0.999
**Digit Span**	14.8 (4.1)	14.9 (3.7)	−0.181	>0.999

^a^Statistics presented: *n* (%); Mean (SD).

^b^Statistical tests performed: chi-square test of independence; *t* test; Bonferroni correction for multiple testing.

^1^Montgomery Åsberg Depression Rating Scale, ^2^Behavioral Activation for Depression Scale, ^3^Beck Hopelessness Scale, ^4^Apathy Evaluation Scale, ^5^Rumination Response Style Scale, ^6^Snaith Hamilton Pleasure Scale, ^7^World Health Organization Disability Assessment Schedule, ^8^General Self-Efficacy Scale, ^9^Mini Mental State Examination, ^10^Digit Symbol Substitution, ^11^Dementia Rating Scale, Initiation/Perseveration.

**Fig. 2:**
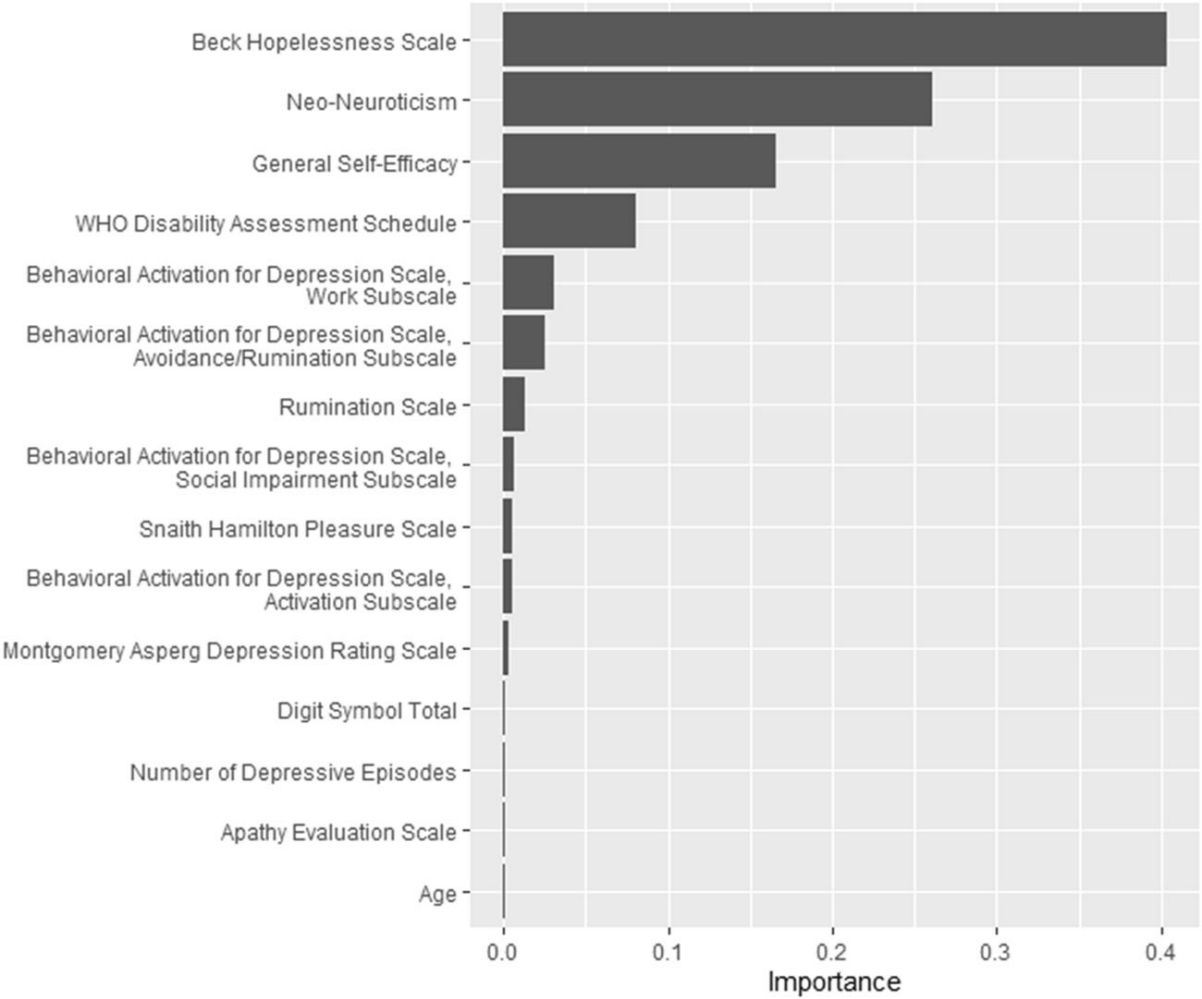
Variable importance in predicting membership to suicidal ideation trajectories in older adults with major depression (*N* = 249) during 9 weeks of treatment with “Engage” or problem-solving therapy estimated by gradient-boosted machine. Predictors are ordered from top to bottom in order of importance in the Gradient-Boosted Machine. The horizontal axis represents the variable importance measure in the Gradient-Boosted Machine that quantifies the improvement in prediction accuracy due to a predictor.

Three machine learning algorithms were used to identify the strongest predictors of membership to the two LGMM of suicidal ideation. LASSO demonstrated the highest predictive performance based on nested cross-validated AUC (0.735, 95% CI 0.67–0.80), followed by the Gradient Boosting Machine (AUC: 0.725, 95% CI 0.66–0.79) and Random Forest (AUC: 0.684, 95% CI 0.6–0.75). Two variables resulted in non-zero coefficients after fitting LASSO on the full data, the Beck Hopelessness Scale (BHS) and the NEO Neuroticism Scale; high scores on both scales predicted membership to the unfavorable suicidal ideation trajectory. These were also the top two most important predictors determined by both the Gradient Boosting Machine (Fig. 2) and the Random Forest, followed by General Self-Efficacy (GSA) scores.

**Fig. 3:**
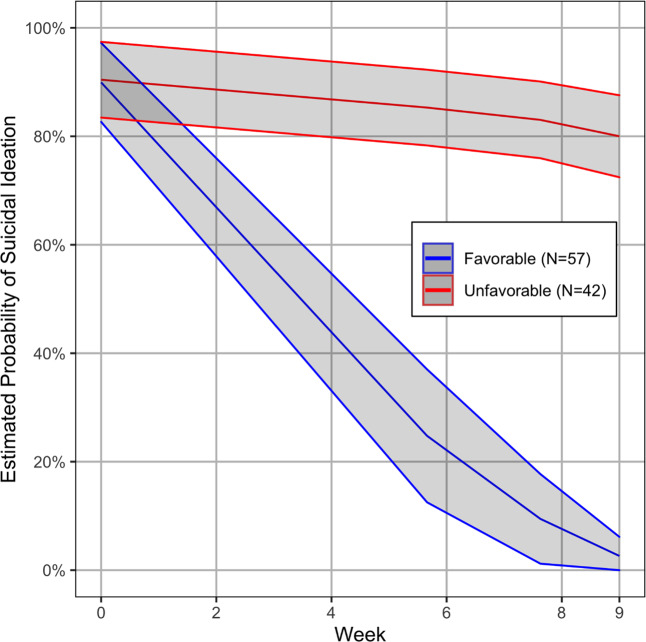
Latent growth mixture model (LGMM) estimated growth curves of the presence of suicidal ideation in 99 older adults with major depression and suicidal ideation at baseline randomly assigned to “Engage” or Problem-solving therapy. The figure presents two LGMM trajectories with 95% CI of the probability of the presence of suicidal ideation over 9 weeks. Red color represents an unfavorable (42% of participants) and blue color represents a favorable trajectory of suicidal ideation (58% of participants). The presence of suicidal ideation at baseline and during the treatment trial is defined as a score of 1, 2, or 3 in the Suicide item of the 24-item Hamilton Depression Rating Scale.

A secondary analysis focused on the subgroup (*N* = 99) that had suicidal ideation at baseline, i.e., HAM-D Suicide Item scores of 1 (feels life is not worth living), 2 (wishes he/she were dead, or any thoughts of possible death to self), or 3 (suicidal ideas or gestures); individuals with score of 4 (attempts at suicide) were exclude from this study. LGMM again showed that the presence of suicidal ideation during treatment was best captured by two trajectories (Fig. 3; lowest BIC among candidate models with 2–5 trajectories), a favorable and an unfavorable trajectory comprising 57 and 42 depressed participants respectively. Again, converging findings of the machine learning analyses showed that high BHS and NEO Neuroticism Scale scores and low GSA scores were the strongest predictors of membership to the unfavorable trajectory of suicidal ideation. Among those with a favorable trajectory of suicidal ideation, 46% had an unfavorable depression severity trajectory. Of those with an unfavorable suicidal ideation trajectory, 17% had a favorable depression trajectory.

#### Classification tree

**Fig. 4:**
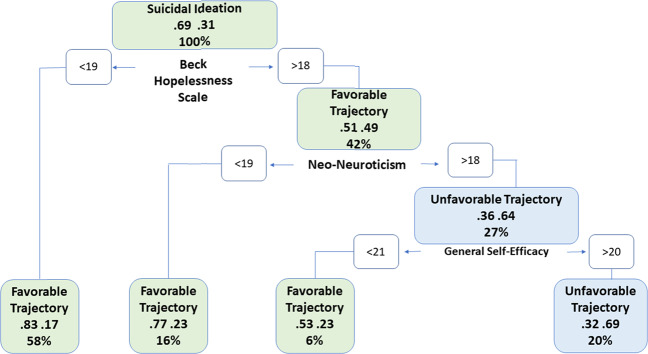
A classification tree predicting membership to the two suicidal ideation trajectories using the three strongest predictors of suicidal ideation in older adults with major depression randomly assigned to “engage” or problem-solving therapy. The classification tree offers a clinical decision rule for identifying membership to either the favorable or the unfavorable trajectory of suicidal ideation during psychotherapy. At each box, participants are classified into either the left or right succeeding box based on the decision rule displayed. Green boxes signify higher probability to have a favorable trajectory of suicidal ideation and blue boxes signify a higher probability to have an unfavorable trajectory of suicidal ideation. The final prediction of trajectory membership is determined by the final box a participant falls into. Each box presents the proportion of total participants who fall into that box (bottom proportion), as well as the proportion of who belongs to the favorable and the unfavorable trajectories (left and right proportions in center of box, respectively), irrespective of the box prediction. For example, the first decision rule split indicates that 58% of participants had hopelessness scores <19; 83% of these participants had favorable trajectories, while 17% of these participants had unfavorable trajectories.

We constructed a classification tree using the three strongest predictors of suicidal ideation trajectories collectively identified by the three machine learning algorithms of this study. Together these three predictors explained more than 80% of the total predictor variable importance of each of the three machine learning algorithms. The resulting classification tree highlights the probability of membership to the favorable suicidal ideation trajectory in participants with combinations of three predictors BHS, NEO Neuroticism, and GSA (Fig. 4). Depressed participants with BHS score lower than 19 had an 83% chance to have a favorable course of suicidal ideation during psychotherapy. Even among participants with BHS greater than 18, those with NEO Neuroticism scores lower than 19 had a 77% chance to have a favorable suicidal ideation trajectory. However, participants with BHS higher than 18 and NEO neuroticism scores higher than 18, had a 69% chance to belong to the unfavorable suicidal ideation trajectory if they had GSA greater than 20. The prediction accuracy of the classification tree (AUC = 0.670, SD = 0.10) was somewhat lower than that of LASSO, the best performing machine learning model (AUC = 0.745).

## Discussion

The principal finding of this study is that the presence of suicidal ideation followed two distinct trajectories during treatment of late-life major depression with either “Engage” or PST. Approximately 31% (76/249) of depressed participants had an unfavorable trajectory of suicidal ideation and 69% (173/249) had a favorable trajectory leading to the absence of any suicidal ideation prior to treatment end. There was only partial overlap of the trajectories of suicidal ideation and the trajectories of depression severity during treatment. Converging information from four machine learning algorithms identified hopelessness, neuroticism, and low general self-efficacy at treatment start as the strongest predictors of an unfavorable trajectory of suicidal ideation. There was no significant difference in the course of suicidal ideation between “Engage”-treated and PST-treated patients. To our knowledge, this is the first study to describe distinct trajectories of suicidal ideation during psychotherapy of late-life major depression and identify modifiable predictors. A small number of potent, modifiable predictors of the course of suicidal ideation can focus clinical assessment and inform the selection of psychotherapeutic interventions.

Hopelessness emerged as the strongest predictor of unfavorable course of suicidal ideation during psychotherapy. This observation is consistent with literature linking suicidal ideation to hopelessness. In a classical study, hopelessness was 1.3 times more likely than the severity of depression to explain suicidal ideation in patients with mood disorders [25]. The ecological momentary assessment showed that, even though hopelessness fluctuates over time, it has a significant association with both passive and active suicidal ideation [26]. Trait hopelessness was shown to have a stronger association with suicidal ideation than hopelessness influenced by depression symptoms [27]. This observation parallels the findings of our study in which hopelessness at baseline, and not the severity of depression, was the strongest predictor of membership to the unfavorable suicidal ideation trajectory.

Neuroticism was another predictor of persistent suicidal ideation in older patients with major depression. Neuroticism is a partially stable trait, characterized by negative affect and increased sensitivity to stress. Neuroimaging studies suggest that neuroticism is associated with impaired functional connectivity of the amygdala with regulatory cortical networks [28]. In large samples, neuroticism was highly correlated with depression in younger and older adults and both the conditions had a substantially overlapping genetic contribution [29]. In addition to its relationship with depression, neuroticism is associated with suicidal ideation [30]. In depressed older adults, neuroticism was a predictor of poor early response to psychotherapy [31], and low remission rates during treatment with sertraline [32].

A low score on general self-efficacy at the outset of treatment predicted an unfavorable course of suicidal ideation. Self-efficacy refers to an individual’s belief in his/her ability to deal successfully with prospective situations and reflects confidence in exerting control over his/her environment. Individuals with low self-efficacy do not believe in their ability to change negative outcomes. Consequently, they may isolate and avoid negative situations rather than attempt to solve problems. In patients psychiatrically hospitalized after a suicide crisis, suicide ideation at the worst time point was inversely associated with self-efficacy related to suicide actions [33]. Individuals reporting current suicidal ideation also reported significantly lower levels of self-efficacy than those reporting no current suicidal ideation. Further, individuals with a history of multiple actual, interrupted, and/or aborted suicide attempts reported significantly lower levels of self-efficacy than individuals with a single lifetime attempt.

The classification tree analysis offers a nuanced clinical view of the relationships among the three strongest predictors of suicidal ideation during psychotherapy (Fig. 4). Depressed patients with low hopelessness had a favorable course of suicidal ideation during psychotherapy. Even among participants experiencing hopelessness, suicidal ideation subsided in those with low neuroticism scores. However, among participants with high hopelessness and neuroticism scores, suicidal ideation persisted in those with high general self-efficacy. So, while general self-efficacy predicts the decline of suicidal ideation overall, it was associated with persistent suicidal ideation in depressed patients with neuroticism and hopelessness. Hopelessness and high self-efficacy rarely coexist. Nonetheless, confidence that a person can take control when he/she believes that nothing can work may make suicide appear as an attractive alternative.

Hopelessness, high emotional reactivity related to neuroticism, and self-efficacy are modifiable clinical factors that, if addressed, may improve the course of suicidal ideation. Identifying themes related to hopelessness and training individuals to examine the validity of their assumptions, have been shown to decrease hopelessness [34]. Emotional reactivity associated with neuroticism may respond to targeted interventions. Brief training in mindfulness meditation can increase self-control and improve the function of cognitive control networks [35]. Mindfulness meditation decreased emotional reactivity, reduced amygdala responsiveness, and increased amygdala-ventromedial prefrontal cortex connectivity during affective stimuli [36]. Identification of patient- and situation-specific triggers of negative emotions and addressing them with cognitive reappraisal and strategies for adaptive responses may reduce negative emotions and suicidal ideation in depressed patients with high neuroticism [37]. Finally, several interventions have been shown to increase self-efficacy. These include setting graded tasks, instruction on where, when, and how to perform desirable behaviors, prompt self-monitoring of behavioral outcomes, motivational interviewing [38], and action planning. Many of the above interventions have been part of both “Engage” and PST. Identifying patients with hopelessness, neuroticism, and low self-efficacy early in treatment and targeting them with appropriate strategies may further reduce suicidal ideation and ultimately decrease the risk of suicide.

The study has limitations. Individuals with imminent risk of suicide were excluded and most participants had mild suicidal ideation. However, mild suicidal ideation is more persistent than severe suicidal ideation and requires clinical attention [39]. Depressed elders with passive suicidal ideation are more likely to have a history of suicide attempts, higher scores of hopelessness [40], slower treatment response, and lower rates of response than non-suicidal depressed elders [39]. Passive suicidal ideation has a stronger association with medical comorbidity and service utilization than active suicidal ideation or no suicidal ideation [41]. Finally, 35% of patients with suicidal ideation change ideator status during the index episode; passive ideators become active ideators and vice versa [40]. Another limitation is the small number of executive function tests of our study. Executive dysfunction is a predictor of poor response of depression to pharmacotherapy [42] and may interfere with problem-solving and the performance of treatment assignments during psychotherapy. Similarly, the study did not assess loneliness, social isolation, bereavement, role transitions, interpersonal conflicts, thwarted belongingness, and other interpersonal factors that had been associated with suicidality [43].

Strengths of the study include its rigorous randomized clinical trial design and the high fidelity of therapists to manualized treatments [7]. The strongest predictors of the trajectories of suicidal ideation were identified through concordance of findings by four state-of-the-art machine learning algorithms. Rigorous estimates of prediction accuracy were obtained by cross-validation to ensure high reproducibility of our prediction model and support the clinical use of hopelessness, neuroticism, and self-efficacy (Fig. 2) as predictors of suicidal ideation during psychotherapy of late-life depression. Finally, classification tree analysis offers a clinical decision rule for predicting the trajectory of suicidal ideation in depressed older adults. However, its findings require replication before introduction into clinical practice.

In conclusion, two trajectories best described the presence of suicidal ideation during psychotherapy of older adults with major depression. While many predictors of suicidal ideation have been reported [43], the convergence of machine learning findings identified three potent predictors (hopelessness, neuroticism, and general self-efficacy) that should not be missed in the assessment of depressed older adults starting psychotherapy. Identifying these predictors early can shape the therapists’ strategy by including interventions targeting hopelessness, emotional reactivity related to neuroticism, and low self-efficacy and improve the course of suicidal ideation in depressed older adults.

## Supplementary information

Supplemental Materials
