# Supplementary material for: Modifiable predictors of suicidal ideation during psychotherapy for late-life major depression. A machine learning approach
Source: Transl Psychiatry. 2021 Oct 18;11:536. doi: 10.1038/s41398-021-01656-5 (PMC8523563; doi:10.1038/s41398-021-01656-5)
Supplement: Supplementary file 1 — Supplemental Materials [file 41398_2021_1656_MOESM1_ESM.docx]

**Modifiable Predictors of the Course of Suicidal Ideation during Psychotherapy for Late-Life Major Depression. A Machine Learning Approach**

**SUPPLEMENTAL MATERIAL**

**eFigure 1.** Flow of Participants in the “Engage” vs. PST Trial

**1,542** Individuals had an initial screen

Phone Screens for Engage

**972** Excluded

**805** Ineligible

- **47** Young age
- **95** Other mental disorders
- **45** In psychotherapy
- **15** On changing dosages of antidepressants
- **245** Low severity of depression
- **252** Ineligible, failed to complete screen
- **106** Other reasons

**167** Eligible, but declined participation

**570** Had Structured Clinical Assessment

**270** Excluded before randomization

- **255** Did not meet selection criteria
- **15** Failed to complete the assessment

**300** Randomized

**149** Allocated to PST

**151** Allocated to “Engage”

**18** Declined participation

**9** Never started PST

**2** Could not be located

**15** Declined participation

**6** Never started “Engage”

**1** Could not be located

**129** Included in primary analysis

**120** Included in primary analysis

**eFigure 2.** Model-based Suicidal Ideation Trajectories: Mixed Model estimated probability of presence of suicidal ideation with 95% CI by treatment arm (“Engage” vs. Problem Solving Therapy) over the treatment period.


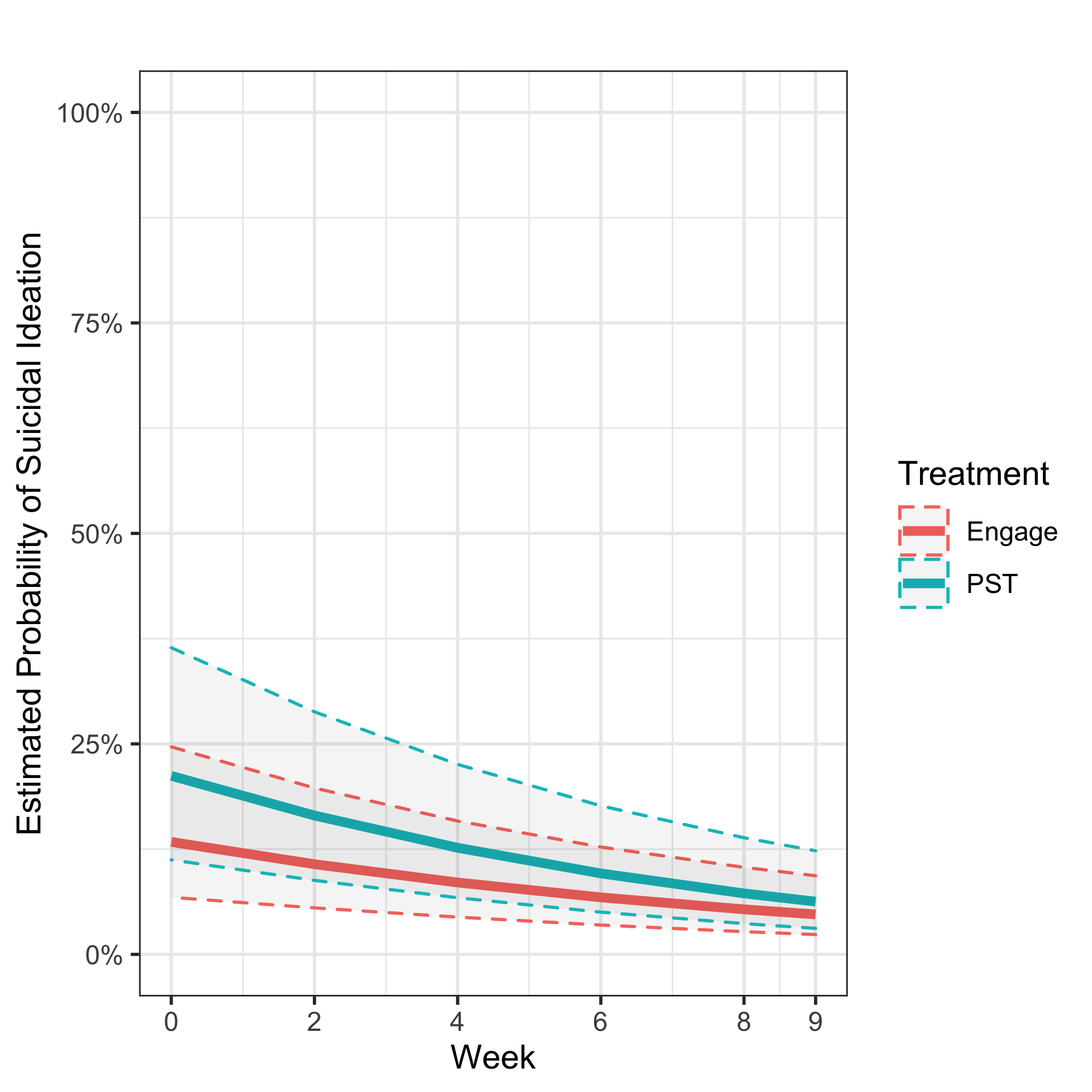


**Legend to eFigure 2.** Presence of suicide ideation is defined a score of 1, 2, or 3 in the Suicide Item of the Hamilton Depression Rating Scale.

**eFigure 3.** Latent Growth Mixture Model (LGMM) estimated growth curves of depression symptoms and signs, except the suicidal ideation, in 249 older adults with major depression randomly assigned to “Engage” or Problem-Solving Therapy.


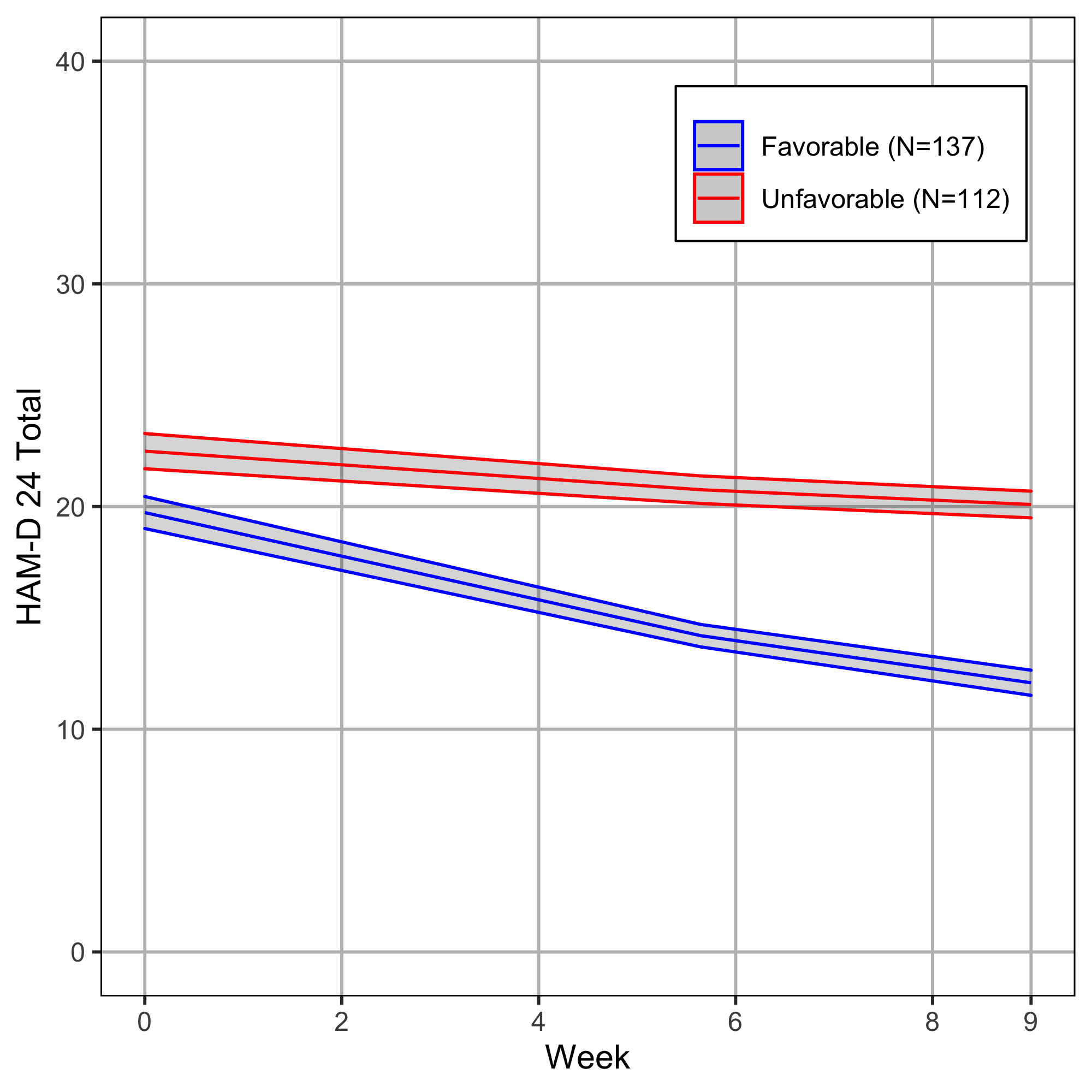


**Legend to eFigure 3.** The figure presents two LGMM trajectories of depressive symptoms other than suicidal ideation (assessed with the 24-item Hamilton Depression Rating Scale (HAM-D) minus the suicide item) along with 95% CI over 9 weeks. Red color represents an unfavorable (45% of participants) and blue color represents a favorable trajectory (55% of participants)..

ENGAGE

Engage in Rewarding Activities:

A Stepped Psychotherapy for Late-Life Depression


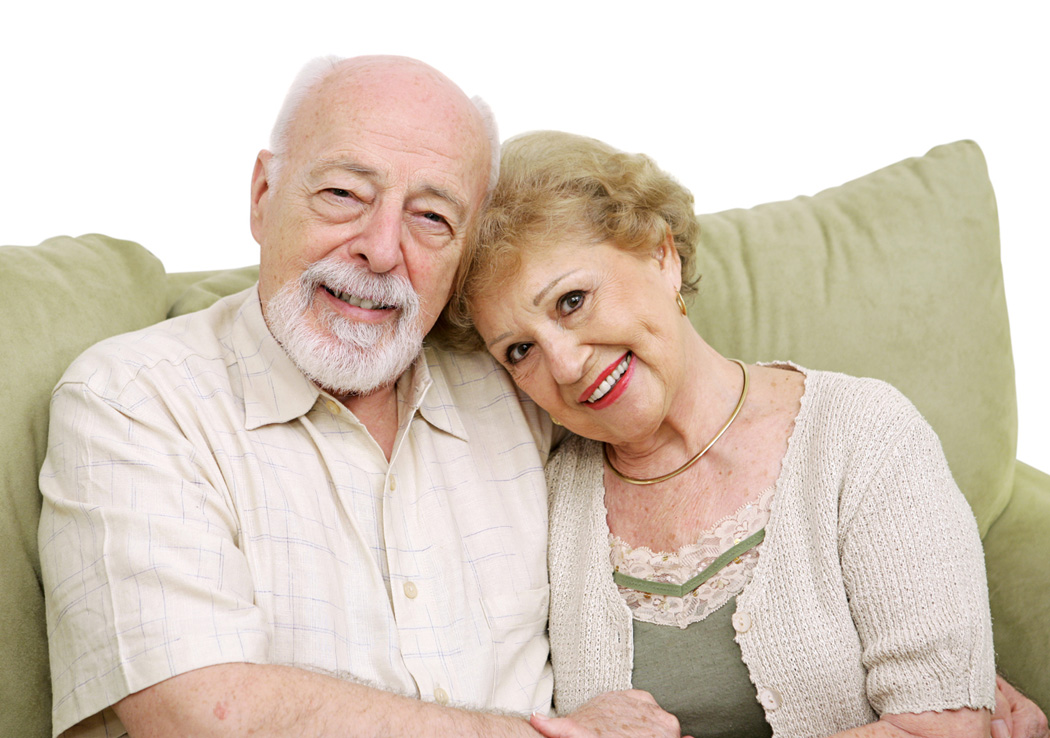


# **Overview**

Welcome to the Engage Manual for late-life depression. Included in the Manual are:

- A guide for helping older adults engage in rewarding activities
- A set of strategies for managing behaviors that serve as barriers to engagement in rewarding activities
- Instructions on how to manage common problems interfering with ENGAGE treatment (e.g., pain, hospitalization, and sleep)
- Session Materials

# **What is Engage?**

# The principal treatment vehicle of Engage is “reward exposure” consisting of reintroducing activities that patients once found rewarding and enjoyed, but have abandoned after they developed depression. Engage uses basic problem solving through which patients learn how to form “action plans” for pursuing rewarding activities of their choice. They are instructed to: 1) identify a goal, i.e., a rewarding and pleasurable activity; 2) develop a list of ideas of what to do in order to meet the goal; 3) select an idea; and 4) create an “action plan” that addresses obstacles that could interfere with successful plan implementation.

The Engage therapist guides patients to select among activities related to social engagement, intellectual exchange, physical exercise, volunteerism, etc. Some patients, however, do not respond to direct “reward exposure.” Common “barriers” to engaging in or deriving pleasure from rewarding activities are:

1. Emotional dysregulation;

2. Negativity bias; and

3. Apathy leading to inertia and inactivity.

Engage uses specific strategies to address each of these “barriers” so that they do not interfere with the development and implementation of “action plans.” However, a good number of depressed older adults can work directly with their “action plans” and engage in rewarding activities without requiring additional strategies. For this reason, Engage follows a stepped approach. It starts with “reward exposure,” a direct attempt to reengage patients in rewarding and pleasurable activities and utilizes additional strategies later, and only if needed following the timetable of the Figure below.


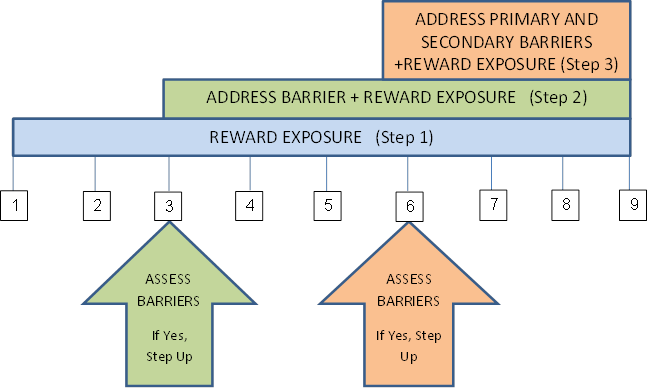


In Step 1, all patients are instructed to identify and engage in rewarding social and physical activities and are taught the “action planning” process. Throughout the first three sessions, Engage therapists assess whether patients: 1) have learned how to form “action plans” pertinent to their needs; 2) have been engaging in rewarding activities as planned; and 3) have begun to show improvement of depression. If all conditions are met, patients continue with Step 1 (“reward exposure”) until the end of treatment.

If the above conditions are not met, therapists review their experience with patients to identify the most prominent “barrier” before the end of session 3. A similar assessment is made between session 3 and the end of session 6. Patients who are doing well with the Step 2 approach (e.g., reward exposure plus strategies for negativity bias) should continue with the Step 2 strategies until the end of treatment. For those who still experience difficulties, the therapist should identify if another barrier exists and add a strategy to counteract it (Step 3). For example, a patient is not engaging in rewarding activities despite the fact that a strategy for his/her “negativity bias” has been used. In this case, during Step 3, the therapist may either use an alternative approach to address “negativity bias” or determine that another barrier (e.g. apathy leading to inertia) is operative and targets this barrier with an appropriate strategy. Strategies for each of the three “barriers” are discussed later in this Manual. Some of the Engage interventions are implemented *during sessions*. Others are applied *between sessions* and are used to facilitate the implementation of “action plans” leading to “reward exposure.”

**Why Stepped Care?**

“Reward exposure” through engagement in rewarding social and physical activities can improve depressive symptoms and signs and disability. Moreover, it is a relatively simple intervention for therapists to deliver and makes sense to many older adults.

Engagement in rewarding activities may not help all patients with late-life depression. Negativity bias, apathy, and emotional dysregulation may inhibit pursuit of action plans and engagement in rewarding activities. These problems may be apparent in the initial assessment of a patient, but emerge most reliably after unsuccessful attempts to engage patients in the “action planning” process.

Diversity in barriers to engagement in rewarding activities requires a targeted approach. Having the ability to add strategies based on patient presentation and response to the initial Engage intervention (reward exposure) personalizes treatment, and can increase the number of depressed patients who can benefit from treatment.

**Why Not Use All Strategies at Once?**

A stepped approach to treatment enables older patients to socialize into psychotherapy, become comfortable discussing their problems, and develop trust in the therapist. Many depressed older adults respond to re-engagement in rewarding activities alone and require no additional interventions. Others have difficulties with multi-component interventions. While it is possible to treat some older people with all available options, a “full-tilt” approach to treatment may not be feasible or acceptable for many depressed older adults, and a stepped approach allows for better treatment personalization.

# **Step 1 – Reward Exposure**


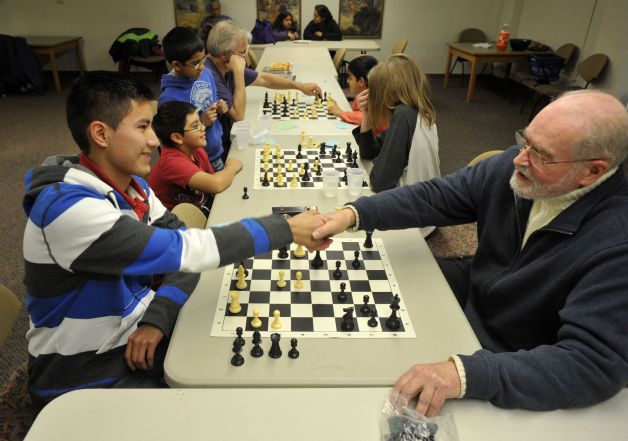


Step 1 consists of three weekly sessions. The goal of the first three sessions is to socialize patients to the treatment and help them engage in rewarding activities using the “action planning” process. Engagement in treatment includes education about depression. Beyond destigmatization of depression and its treatment, the therapist has the opportunity to explain how depression can lead to social isolation and lack of interest in once rewarding activities. Patients are made aware that engaging in activities can improve mood, and as a result help one feel energized and able to take on bigger problems. The therapist also engages the patient in a discussion about goals for change and problems that may emerge.

***Creating a supportive environment.*** Engage therapists create and maintain warm, trusting, and supportive relationships with patients. They convey concern for patients and their lives and approach their experiences and feelings with empathy. As in other psychotherapies, the therapeutic relationship in Engage is seen as a condition necessary for therapy to proceed. For this reason, therapists must be attuned to the patients’ point of view and be aware of the degree to which patients agree on the goals and tasks of Engage. If problems emerge or if patients express disagreement on the proposed approach, therapists should address their concerns and attempt to resolve disagreements.

***Session Structure.*** Engage is a semi-structured intervention. Early sessions cover a good deal of material, while later sessions will be less content heavy and focus mostly on helping patients implement the agreed upon activities. Regardless of content, the therapist should structure each session in the following way:

- Set an agenda: Let the patient know what you need to cover, and then ask the patient if there are other items to add to the agenda. Structure the agenda in this way:
  - Administer the PHQ-9 (a depression rating scale)
  - Develop or review the patient’s “action plan”
  - Create a new “action plan”
- Order and prioritize the session content, except during crises. If the patient is in a crisis (e.g., an upsetting event), devote the first part of the session to addressing the crisis.
- Redirect patients when needed. Some patients are easily distracted. Let those patients know that you will try to help them follow the agenda and remind them to do so when needed.
- Use the Action Planner in the session.
- Summarize the session at the end and ensure that patients are clear about their goals and understand how to use their “action plan.” Give patients a copy of their “action plan” as a reminder of what they decided to do during the week.

***Session 1***

Session 1 has three aims:

1. Socialize the patient to *ENGAGE*;
2. Work with the patient to make a list of rewarding social and physical engagement goals;
3. Develop an “action plan” with the patient consisting of 2-3 activities that the patient would like to pursue between sessions.

To socialize the patient to Engage*,* the therapist first explains the structure of treatment, specifically that they will meet together for 9 sessions lasting 40-50 minutes each. The therapist briefly reviews the patient’s initial PHQ-9 score, most distressing symptoms, and duration of depression. The therapist discusses the importance of weekly PHQ-9 ratings as way to monitor symptoms and the patient’s response to treatment.

The therapist then educates the patient about the connection between social and physical activities and mood, and how it is important to remain active and positive. The therapist explains how the patient and therapist work together in Engage to set weekly social and physical goals by creating action plans in session. The therapist highlights the importance of the patient following through with these action plans out of session, and creating new action plans on the his or her own. The therapist explains that they will review the patient’s success or difficulties in completing their actions plans every session. They will identify any barriers to re-engagement the patient experiences so that the therapist and patient can work together to develop strategies to overcome these barriers. The therapist then reviews expectations the patient has about treatment and addresses any concerns about treatment.

The second aim of Session 1 consists of asking the patient about changes in activities due to changes in health, life circumstances (e.g., new caregiving responsibilities), and mood. This discussion is meant to answer the following questions:

1. What activities has the patient dropped and would like to resume?
2. Are there any health concerns or goals the patient has?
3. Are there activities the patient hoped to be pursuing at this stage in life but has not?
4. Are there barriers to achieving the patient’s goals?

Based on this discussion, the therapist creates a list of activities that the patient might find rewarding or pleasurable (see Session Materials). The therapist asks the patient to rate how easy or hard they are to pursue, and gives the patient a copy of this list.

After this discussion, the therapist introduces the *Action Planner* and uses this tool to help the patient create a plan for pursuing one or more activities during the ensuing week. For these first three weeks, the selected activities should be simple and easy to achieve.

***Creating Action Plans.***

**Action Planning**

1. Select a goal 5. How did it go?

2. Develop ideas for meeting the goal 6. Identify barriers

3. Choose an idea

4. List steps of action plan

Completing the Action Planner involves the following steps:

1. Select a goal. The goal for the week should focus on a social activity, a physical activity, or some other activity that the patient would like to pursue, but has been unable to do so. Examples of goals are:

**Social Goals**

Spending more time with family/friends Volunteering

Meeting new people Working

Traveling Resolving a conflict

**Physical Activity Goals**

Exercising more (specific exercise) Losing weight

Gardening Sports

Finding a Hobby Managing an illness

**Other Activity Goals**

Financial Spiritual

Legal Educational

Hoarding/Clutter

The goal should be clear and succinctly defined, so that the discussion about ways for reaching that goal does not go too far afield. We suggest that in the first three sessions, the focus be on social and physical goals, as the discussion around other goals can be challenging while the patient is still feeling depressed.

2. Develop ideas for meeting the goal. Once the goal has been set, the therapist helps the patient generate ideas to reach the goal. Depressed people often have a difficult time generating ideas, partly because they are discounting the value and effectiveness of their ideas before adequately defining them. Teaching individuals to creatively think of a range of possible ideas is based on the premise that the availability of many alternatives increases the chances of identifying effective ideas. In other words, the *first idea* that comes to mind is not always the *best idea*. Therefore, it should be emphasized to patients that they should try to generate as many ideas as possible via *brainstorming* techniques. Additionally, ideas should be clear and concise. For instance, if the goal is to socialize with friends more, ideas should be along the lines of “Go to the movies this week with a specific friend,” “Go to a church social,” or “Talk to a friend on the phone.”

It is important that the ideas come from the patient. Sometimes this is difficult for depressed patients to do. If patients cannot generate ideas:

- Ask patients if they are having trouble because they cannot think of ideas or because the ideas that are coming to them are not appealing. If the latter, tell them to write down the ideas, as they can be adjusted to seem more attractive if discussed. You may also ask them what they would recommend to a friend, or what others might recommend to them.
- If patients still have difficulties generating ideas and solutions, you may offer to give them a set of ideas other people have proposed.

3. Choose an idea. Once you have a list of ideas, have patients evaluate them by asking the following questions:

- Is the idea achievable with a reasonable amount of effort and time?
- Could the patient see him/herself pursuing this idea?
- Does it cause other problems?
- Will it meet the patient’s goal?

Based on how the patient answers these questions, the most sound idea will emerge. Sometimes, all ideas are sound and it will be up to the patient to make a judgment about which idea to try first. The session then should focus on planning for its implementation. If the patient’s ideas are unrealistic, you may either have the patient propose more ideas or pick the most sound idea and discuss with the patient how to improve it.

4. List steps of action plan. Once an idea is chosen, the next step is to talk with the patient about specific steps necessary to implement it. This is an important part of the “action plan,” because many people can generate good ideas on how to meet a goal, but have problems implementing them. This is because once the time comes to implement the idea, patients feel overwhelmed and may even be unclear where to start. Therefore, it is useful to ask patients to describe how they envision implementing their plan. It can be helpful to ask patients to close their eyes and picture themselves engaging in a step-by-step implementation of the plan they selected. Have them then consider:

- Who would be involved?
- Where will the activity take place?
- Is there anything they will need to prepare for the activity?
- When is the best time to start?

Once patients have considered all they need to implement their idea, help the patient list specific steps of the “action plan”. As an example, going to the movies with a friend involves the following steps:

- Calling friends to see when they are available
- Picking a movie and movie time
- Arranging transportation to the movie
- Making sure they have money for the movie.

5. How did it go? After patients implement the plan, they will need to assess how it went and how they felt after they implemented the plan. We provide a visual method for rating the plan, but patients can record their success however they see fit, as long as they evaluate how the plan went.

6. Identify Barriers. Forewarn patients that sometimes plans may not work out at first. Reassure them by indicating that understanding barriers to the plan’s implementation can help modify the plan so that it addresses their own needs. Have them record any barriers; these could be time constraints, feelings of resignation, lack of motivation, or other medical illnesses or disabilities. Discuss these barriers in session and help patients make more effective plans.

***Session 2***

The purpose of Session 2 is to continue helping patients develop “action plans” and guide them in seeing the connection between re-engagement in rewarding activities and improvement in depressive symptoms. The therapist uses the Action Planner and the PHQ-9 scale, but also keeps a careful eye out for three common “barriers” to successful implementation of planned rewarding activities:

- Emotional dysregulation
- Negativity bias
- Apathy leading to inertia

It is useful to distinguish whether failure to engage in a planned rewarding activity is due to unexpected events or originates from one of the above three “barriers.” See Step 2 for more details on how to recognize these “barriers.” In the initial phases of Engage (session 1 and 2), it may not be necessary to intervene. In addition to identifying and evaluating barriers, it is important to review changes in PHQ-9 depressive symptoms.

***Session 3: Decision-making***

Before the end of Session 3 (but in some cases in Session 2), the therapist decides if the patient would benefit by continuing to work on Step 1 or needs to move to Step 2. This decision is based on a combination of factors including:

- Engagement in selected rewarding activities
- Ability to complete “action plans”
- Ability to retain focus during sessions
- Changes in PHQ-9 scores
- Subjective patient report of improvement
- Clinical judgment

Criteria for continuing with Step 1 are: a) engagement in selected rewarding activities; b) ability to complete “action plans” despite difficulties with emotion management, negativity bias, and apathy leading to inertia; and c) improvement in mood and functioning (>30% reduction in session 1 PHQ-9 score). Patients continuing with Step 1 should be re-evaluated in another three weeks. Patients who are struggling with “action plans” because of emotional dysregulation, negativity bias, or apathy should move to Step 2.

Use the Barrier Rating Scale (see Session Materials) to identify primary barrier.

***Step 1: Sessions 4-9 (For patients without barriers to “action plans”)***

Continue to work on one to two additional goals for engagement in rewarding activities, and look for the following cues to identify whether the patient is improving:

1. Use of Engage: Optimally, patients should begin to effectively use the action planning process on their own.
2. Improvement of depression: Use the PHQ-9 to assess improvement of depression. A good number of patients may have 50% reduction in their PHQ-9 scores by week 6 or 7. Some patients may report noticeable improvements in mood and function not reflected in the PHQ-9.

Patients who do not continue to show improvement between Sessions 3 and 6 may move to Step 2 by Session 6.

Use the Barrier Rating Scale (see Session Materials) to identify additional barrier.

***Preparing for termination:*** By Session 8, the therapist should begin discussing treatment termination. Start Session 8 by reviewing progress made in functioning and social and physical activation since the beginning of treatment. Use the Relapse Prevention Planner (see Session Materials) to develop a prevention plan. The relapse prevention plan includes:

1. Early warning signs of an emerging depression;
2. Activities having positive impact on mood;
3. Education on symptoms and signs of depression requiring medical attention.

***Step 1: Last Session***

In this session:

1. Review the relapse prevention plan;
2. Provide additional copies of the PHQ-9 mood tracker and Action Planner;
3. Congratulate the patient on completing the Engage program.

**Step 2 – Adding Strategies**


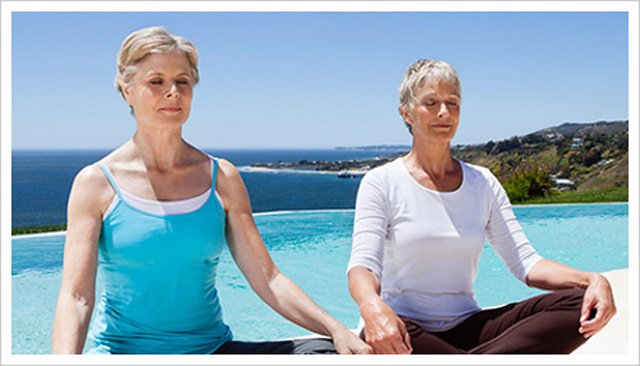


This section covers strategies for overcoming depression-related barriers to successful treatment. Specifically, it covers how to decide on which barrier to focus, strategies you can use in-session, and strategies to teach Step 2 patients for managing negativity bias, apathy leading to inertia, and emotional dysregulation.

**Deciding to Move to Step 2**

The decision to move to Step 2 should be personalized based on three indicators:

1. *Improvement of depression:* Although 3 weeks may seem too early to make a judgment about treatment response, many patients begin to show some improvements in mood and other symptoms by that time. Move to Step 2 for patients who do not show >30% reduction in session 1 PHQ-9 score at session 3.
2. *Ability to complete “action plans” and engage in rewarding activities:* Ability to create and implement “action plans” is a good sign even when improvement of depression is lagging. Examine whether patients can select reasonable goals, identify feasible strategies to meet them, and implement their “action plans.” Patients who do not fully complete them should be considered for Step 2, even if their PHQ-9 trajectory indicates some improvement in depression. The rationale is that improved mood in the absence of behavioral change is a risk for relapse.
3. *Behavior in session:* Even though a patient may understand the action planning process and their depression score is improving, the therapist may notice that implementation of “action plans” lags because of excessive emotionality. Another patient may understand the first step of Engage and formulate “action plans,” but find them irrelevant to their problems because of a “negativity bias.”

**Deciding on Which Barrier to Focus**

As discussed above, there are three common barriers to engagement in rewarding and pleasurable activities: negativity bias, apathy leading to inertia, and emotional dysregulation. The *Barriers Rating Scale* (see Session Materials) is designed to help determine the barriers a patient is experiencing, and should be completed at the end of Session 3 and again at the end of Session 6. If there is more than one barrier, the Scale will guide the therapist on where to focus during Step 2. Be aware that behaviors associated with these barriers often overlap, and it may not always be easy to identify the barrier interfering with treatment.

***Emotional Dysregulation***

Emotional dysregulation results from inadequate capacity to regulate emotions, and as a result emotions spiral out of control, change rapidly, and overwhelm reasoning. Patients with this presentation will often describe themselves as intense or emotional people, and may be tearful or excessively anxious during sessions. They will have difficulty focusing on discussions of problems or engagement in activities and will often begin talking about past injustices and painful memories. Such patients may benefit from strategies for controlling the impact of their emotions on goal-directed behavior. What follows are signs that the therapist should look for to determine whether emotion management difficulties are barriers to treatment success.

***In-session signs:***

- Does the patient become emotionally overwhelmed to the point that you cannot maintain a treatment focus?
- Does the patient become easily anxious, angry, or sad while talking about problems or activities?
- Does the patient recall memories from the past that become emotionally overwhelming?

***Between-session signs:***

- Are “action plans” not initiated or completed because the patient becomes too anxious or upset while pursuing them?
- Does the patient report continued problems with controlling emotions even when engaged in activities?

***Negativity Bias***

Negativity bias refers to selective attention to negative rather than positive information and experiences. On a behavioral level, negativity bias is evident:

- When given positive information and negative information about another person, the patient’s net judgment about this person is negative, rather than balanced.
- If a patient has a good experience and a bad experience close together, he/she will feel upset and will discard the good experience.
- Negative information has greater impact and attracts more attention than positive information of similar weight. A positive event receives less attention than a negative event.
- When put in a novel environment, a person immediately notices the threats instead of the opportunities.

***In-session signs***

- Does the patient only focus on negative aspects of a situation and seem unable to consider an alternative perspective?
- Does the patient’s negativity interfere with his/her ability to keep treatment focus?
- Is the patient unable to consider or independently generate positive approaches to meeting his/her goals?

***Between-session signs***

- Are “action plans” incomplete because the patient did not believe that they could be effective?
- Are “action plans” incomplete because the patient thought of reasons not to engage in the plan?
- Does the patient fail to value the implementation of an “action plan” because another problem occurred during the week and overshadowed the impact of his/her success?

***Apathy Leading to Inertia***

Apathy refers to lack of motivation interfering with initiation of activities. Apathetic patients quickly loose interest and cannot maintain their focus on the task at hand.

***In-session signs:***

- Is the patient easily distracted?
- Does the patient go off on tangents when talking about goals?
- Does the patient seem disorganized?

***Between-session signs:***

- Are “action plans” incomplete because the patient did not start them?
- Are “action plans” incomplete because the patient was distracted during the week?
- Does the patient fail to use the Action Planner for activities other than those discussed in session?

**Strategies for Emotional Dysregulation**

Provide the patient with the pamphlet “Coping with Feelings”.

Strategies for emotional dysregulation can be anything that helps calm emotions, redirect attention away from unpleasant feelings, and promote relaxation.

**Emotional Dysregulation Strategies**

Meditation Relaxation Exercises Deep Breathing Prayer Imagery

Selection of an appropriate strategy should be based on previous experiences helpful to the individual patient. Therapist and patient should weigh the pros and cons of each strategy. The selected strategy should be practiced first in session and then between sessions so that patients master these techniques and can employ them when they begin to feel overwhelmed. To this end, we recommend that patients find a quiet time and place to practice the selected emotion management techniques for about 10-15 minutes a day. Patients should record their mood before and after practice. They should also be instructed to incorporate these strategies before each step of the “action plan”. Below is an example:

*Ms. M is a 75 year depressed woman who complains of being socially isolated. She has picked as her goal to call an old friend she is comfortable talking to and go out for coffee. When she tried to implement this plan, however, she became overwhelmed with doubt, anxiety, and sadness; these feelings prevented her from picking up the phone to call her friend. Ms. M and her therapist agreed to practice imagery exercises to help her through her intense emotions when she implements her plan. The Engage therapist instructed Ms. M to imagine a pleasant, relaxing scene (for her it was laying on a warm beach). Ms. M even brought in a picture of one of her favorite vacations to the beach to help her visualize the scene. Ms. M and the therapist used this imagery exercise to help Ms. M manage her excessive emotionality during Engage sessions. Below is Ms. M’s new action plan:*

Plan: Who is involved? *Myself and my friend Jane.*

Where will it happen? *I will call Jane on Monday at 2PM, and will set up a time to have coffee, e.g. on Tuesday in the morning, around 10-11 am.*

When will you start? *I will get ready for the call by first thinking about the beach to help me relax.*

What do you need? *Jane’s phone number, a phone, my script, and my beach picture.*

Steps:

- *Spend 5 minutes looking at my picture, then close my eyes and relax*
- *Dial up Jane*
- *Ask Jane right away for coffee*
- *On Tuesday, before the coffee meeting, practice with beach picture to calm my nerves.*
- *Meet Jane*

After you determine that non-response to treatment is due to emotion management difficulties, you will need to discuss your observations with the patient. The feedback should be structured using what is called *“The Feedback Sandwich.”* The feedback sandwich starts with positive observations, continues with discussion of challenges, and ends with positive “action plans.” This method of providing feedback has been found to be helpful in a various forms of communication, and therapy is no exception. For example:

*Ms. J, it is time for us to review your progress together. I want to start by saying that I see you really trying hard to overcome your depression, and you come up with creative ideas for doing so (positive feedback). I also notice that when we talk about your “action plan” you become overwhelmed when you are trying to think of ideas to get more engaged. I have also noticed the same when you try to make an “action plan.” Do you agree? Does that sound like the main problem to you (discussion of the challenge)? This is common in many people with depression. I think together, we can come up with a way to help you get a better handle on your feelings so that they do not get in the way of you feeling better and getting more connected with people. There are a number of strategies that are helpful, let’s talk about the pros and cons of each (ending with positive plan).*

The next step is to generate a set of strategies and select among them those that the patient feels most comfortable using. For some strategies, you may have to teach the patient how to use them in the initial session of Engage Step 2. A training guide for such strategies appears in the Session Materials section. Strategies requiring initial patient training are:

- Relaxation Exercises
- Mindfulness Meditation
- Deep Breathing

Prayer and imagery are relatively easy to do, but may still require practice. Patients will need to practice their chosen strategies between sessions (at least 10 minutes daily) and use them when they complete their “action plans.” See Step 2 session structure for how to incorporate these strategies into the session structure.

**Strategies for Negativity Bias**

Provide the patient with the pamphlet “Coping with Pessimism”.

Strategies for coping with negativity bias help patients evaluate their situation in a balanced way, recognize when they are excessively focusing on negative information, and draw their attention to neutral or positive aspects of the situation. In Engage, we use modified cognitive-behavioral strategies to overcome this barrier. However, instead of applying these cognitive behavioral strategies in all situations, we ask patients to use them specifically when they encounter difficulties in their “action plans”.

Choice of one of the following 3 strategies depends on the patient’s preference and your clinical judgment.

**Negativity bias strategies**

Devil’s Advocate

Weighing the Evidence

Practice Having a Positive Focus

***Devil’s Advocate:*** The Devil’s Advocate technique is one way to help patients counteract their negative thinking. The following are instructions to patients:

1. Write down the reasons for *not* using your “action plan”;
2. Think of the opposite view: What would you say to get someone else who is making the same negative argument about following through with the “action plan”?
3. Organize the positive arguments and refer to them whenever you feel like abandoning the “action plan.” You can also use the Argument Worksheet in the Session Materials section.

***Weighing the Evidence:*** Using the Weighing the Evidence worksheet in the Session Materials section, ask patients to:

1. Estimate the probability that their worst expectations will come true.
2. Think back of all the times they engaged in a similar activity and ask them to recall how often they actually experienced the expected negative outcome. Estimate if that was less or more than half the time.
3. Engage in the “action plan” and then document if the negative outcome actually occurred. Point out to the patient the difference between their expectations and the actual outcome.

Here is an example:

*Ms. B is a 68 year old woman with chronic depression and clear negativity bias. Her “action plan” had been to go to her ballroom dancing classes on a regular basis, not just when she felt like it (she almost never went). She indicated that she was too depressed to go to ballroom dance, and told her therapist that she had a lousy time each time she felt like this. The therapist said, “So you believe that there is a 100% chance of you having a bad time if you went to class today?” to which Ms. B agreed. Then, the therapist said to Ms. B: “Think back of all the times you went to class despite feeling depressed. Did you always have a lousy time? Did you ever have a good time?” Ms. B said that she did not always have a bad time, sometimes dancing distracted her from her depressive thoughts and she did feel better after class. Ms. B and her therapist then agreed that if she went to class that day, she would have a 75% chance (but not 100% chance) of having a lousy time. Her “action plan” was to go to class and see how it went. Ms. B and her therapist agreed that she would stay in class for at least ten minutes, and if she was having a lousy time, she could tell her instructor that she had a headache and leave class. In the week after this session, Ms. B went to class, and reluctantly reported that she was able to enjoy it.*

***Practice Using a Positive Focus:*** Having a positive focus is the brain’s tendency to seek out and pay particular attention to pleasurable and positive cues. Positive thinking is muted during depression, and depressed patients need to work hard to restore it. Patients who chose to counteract their negativity bias by engaging in positively focused thinking may benefit from practicing the strategies listed below until they feel comfortable with them and are able to make them part of their “action plans.”

- **Think outside yourself.** Imagine trying to give other people reasons to pursue your “action plan.” Think about less negative statements that offer more realistic expectations about your “action plan’s success.
- **Keep a “negative thought log.”** Jot down in a notebook any negative thought that comes up while implementing an “action plan.” Review it from time to time and ask yourself if your negativity was truly warranted. Ask a friend or me to go over your log with you so that you can have a second opinion.
- **Changing perspectives.** Review your negative thought log. Then, for each negative thought, write down something positive or find an alternative explanation. For instance, “My boss hates me. He gave me this impossible task to do.” could be replaced with “My boss must have a lot of faith in me to give me so much responsibility,” or “My boss has given me so much work because this is an important project with a short deadline.”
- **Socialize with positive people.** Notice how people who always look on the bright side deal with challenges, even minor ones. Then consider how you would react in the same situation. Even if you have to pretend, try to adopt their optimism and persistence when implementing your action plan.

**Strategies for Apathy Leading to Inertia**

Provide the patient with the pamphlet “Improving Concentration and Disorganization”.

The very process of creating an “action plan” can help depressed patients with mild apathy. However, patients with significant apathy may need to add prompts for initiating their “action plans.” Examples of such prompts include:

- Checklists for complex “action plans”;
- Signs and equipment necessary for daily activities in full view in the patients’ homes;
- Labels and electronic devices to signify the time to initiate an “action plan”;
- Calling the patient during the week to prompt the “action plan”;
- Involving family and friends in the “action plan” as prompts to initiate it;
- Starting the “action plan” in session.

Apathetic patients often lose their focus soon after they start implementing an “action plan.” Most of the work for managing the loss of focus needs to happen in session. The therapist should work with the patient to create cues that can be used to redirect the patient back onto task. Strategies we have used effectively are:

- “Time out” signs to interrupt patients when they go off into tangents;
- Ask patients if what they are talking about is related to achieving their goal.

At home, patients who lose focus easily may benefit from the removal of distracting items from their environment, e.g., designating one place at home where they can review and complete their “action plans” and other between-session work.

**Step 2 Session Structure**

Sessions 4-6 are similar to each other and use the same session structure detailed in Step 1, but also include:

1. Selecting and practicing the barrier strategies;
2. Including strategies for overcoming barriers in the “action plan.”

***Step 2: Session 4 and 5***

Session 4 begins with a discussion of your observations about barriers interfering with implementation of “action plans.” Then identify strategies to address the barrier the patient and you believe interferes most with implementation of “action plans.” Some strategies (emotion management and negativity bias) require some practice. Leave 10 minutes at the end of session to add the selected strategy to an existing “action plan.” The new “action plan” will consist of:

- The goal and the plan for accomplishing it;
- The strategy to overcome the barrier to plan implementation;
- Time for the patient to practice those strategies.

Provide the patient with the appropriate Step 2 pamphlet.

Session 5 focuses on the implementation of the new strategies and “action plans.” You should continue to observe whether the patient now becomes able to better implement the “action plans” and shows improvement in mood.

***Step 2: Session 6, Decision-Making***

By the end of this session, the therapist decides whether the patient can benefit by continuing Step 2 treatment or needs to move to Step 3.

*Criteria for Continuing in Step 2:* Engage offered according to Step 2 should continue unchanged, if the following conditions are fulfilled:

1. Has the patient begun to use “action plans” on his/her own?

2. Did the patient benefit from the Step 2 strategies to overcome his/her barrier to forming and implementing “action plans”?

3. Does the patient improve by >30% in his/her session 1 PHQ-9 score by week 6?

4. Does the patient report noticeable improvements in mood and functioning?

Patients who do not show such improvements should move to Step 3. Step 3 consists of techniques targeting barriers in addition to those used during Step 2. Step 3 techniques are selected from the same list of strategies for Step 2. However, they should be other than the strategy used in Step 2 that was unsuccessful.

***Step 2: Sessions 7-9, Continuing with Step 2 strategies to overcome barriers***

Continue to use the Action Planner in sessions 7 through 9, work on one to two additional goals each week using the selected technique for barrier mitigation during Step 2. By session 8, it is time to begin discussing termination and relapse prevention. Additional tasks for Session 8 and for the final 9^th^ session are the same as those described above in Step1 Section (under Preparing for Termination and the Last Session).

# **Step 3 – Adding More Strategies**


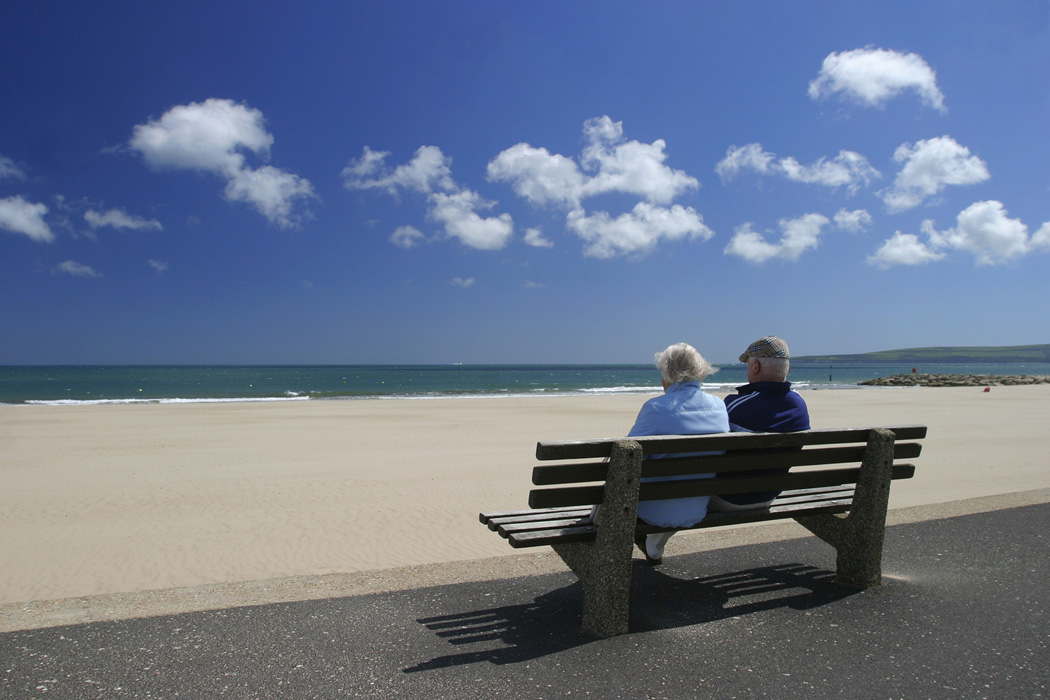


**Deciding to Add Strategies at Step 3**

As discussed above, the decision to move to Step 3 is made by the end of Session 6. By this time, the therapist is in good position to judge what additional barriers the patient is facing when implementing or failing to benefit from “action plans.” If “action plans” are created thoughtfully and strategies to overcome behavioral barriers are mastered, patients should be able to implement and benefit from their “action plans” by six weeks. Patients who fail to complete them partially or fully should be considered for Step 3 strategies, even if their PHQ-9 score is dropping. The reason is that improved mood in the absence of behavioral change is likely to be temporary.

**Selecting a Barrier in Step 3**

The process for selecting barriers to target in Step 3 and the selection of strategies to address them is the same as in Step 2. Use the Step 3 Action Planner to add strategies to the treatment plan and help the patient practice these strategies during each session.

**Step 3 Session Structure**

***Step 3: Sessions 7-9***

At the beginning of Session 7, the therapist discusses with the patient observations about additional barriers interfering with implementation of “action plans.” Together, therapist and patient decide on additional strategies and integrate them into the “action plans.” The new “action plan” now consists of:

- The goal and accompanying plan;
- The additional strategy targeting the most common barrier to implementation;
- Time for the patient to practice those strategies.

Provide the patient with the Step 3 pamphlet.

Continue to use the Action Planner in sessions 7 and 9. Work on one to two additional goals each week using the selected technique for barrier mitigation during Step 3. Additional tasks for Session 8 and for the final 9th Session are the same as those described above in Step1 Section (under Preparing for Termination and the Last Session).

**Does the Patient Need a Different Type of Care?**

This question should be asked throughout treatment and for all patients regardless of the Engage Step required. However, a definite decision should be made by session 8 and shared with the patient. Some patients will need a different type of care, be it antidepressants, a different kind of psychotherapy or other treatments. This way by session 9, the therapist will have a treatment plan and potential referrals to appropriate providers and services.

**Chronic pain, sleep, and hospitalization**


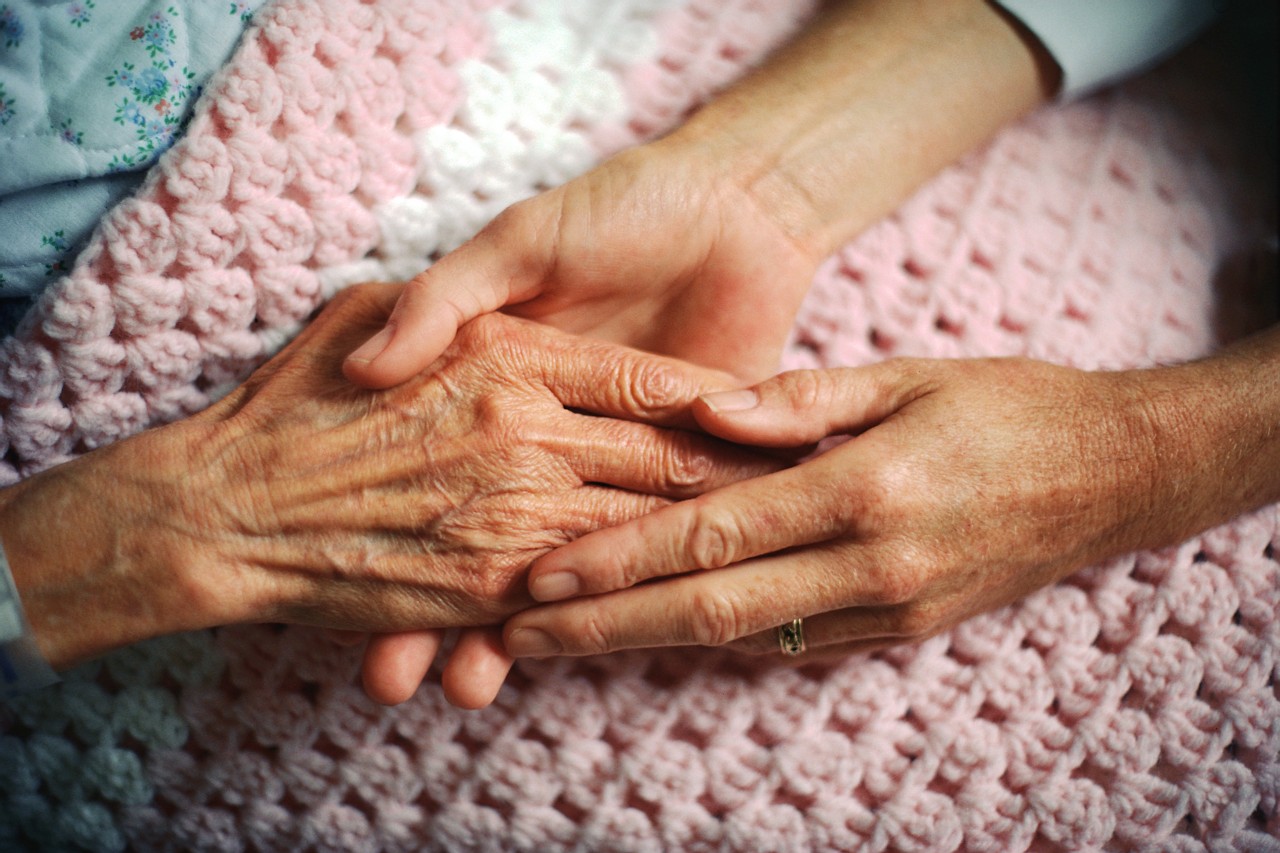


This section briefly covers other barriers to successful treatment and how to address them. Pain, sleep and medical illnesses are all common in late life and may interfere with the success of any depression intervention. We briefly discuss each barrier below and their management during Engage.

***Chronic Pain*:** Patients who had no medical evaluation for chronic pain management should receive appropriate referrals. If a patient has been evaluated and treated but still experiences pain, the therapist may integrate chronic pain management strategies into the Engage treatment plan so that pain does not interfere with the implementation of “action plans.” If a patient avoids an “action plan” on the days in which the pain is most intense, the therapist should plan for “pain day” alternatives, i.e., activities that the patient can pursue despite being in pain.

***Insomnia*:** Sleep difficulty is another common problem in late-life depression. Like chronic pain, insomnia patients may benefit from a sleep evaluation, as some may have sleep problems in addition depression-induced insomnia (e.g., sleep apnea). If the insomnia is mainly related to depression, there are a few sleep hygiene strategies that can help (see Session Materials section). The therapist should encourage patients to use these strategies, as better sleep will give the patient more energy to pursue their “action plans.”

***Illness/hospitalization*:** Hospitalization can interrupt therapy. If the hospitalization is brief and does not require a lengthy post hospitalization recovery period, you should be able to pick up treatment where you left off. However, if the hospitalization or recovery period lasts more than three weeks, you may have to start Engage all over again. Remain in contact with the patient while they are in the hospital to maintain the therapeutic rapport; chances are, they will need your support.

# **Session Materials**


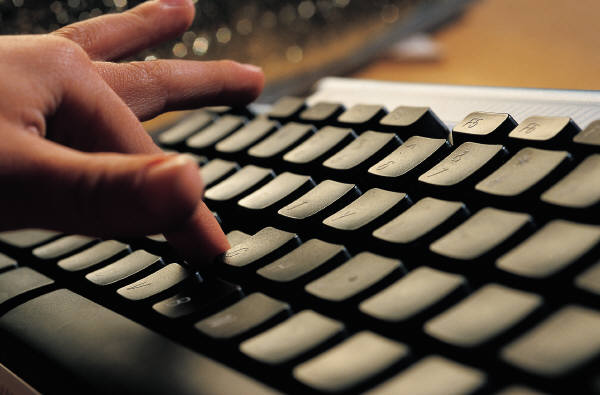


**Engage Therapist Session Note**

Therapist: _________________________ Patient ID:___________________________

Date:_____________________________ Step Number: ______ Session#: ______

**Materials Given:**

- Action Planner
- Step 1 educational materials
- Step 2 or 3 educational materials

Emotion management difficulties

Negativity bias

Apathy leading to inertia

**Severity Ratings:**

- - PHQ-9 Total Score: _____ Sleep Score: _____ Suicide Score: _____

**“Action Plan” review:**

- N/A, session 1
- Primary action plan completed, additional plans created
- Primary action plan completed, no additional plans created
- Primary action plan partially completed
- Primary action plan not completed

Too sick

Too distressed

Didn't think it would help

Too many competing demands

Forgot

**New “Action Plan”:**

- Activity discussed and planned: _____________________________________________________________
- Activity discussed but not planned: __________________________________________________________
- No Activity discussed or planned. Explain: ___________________________________________________
- Termination session; discussed goals and completed relapse prevention plan

**Barriers—only fill out if step 2 (one barrier) or step 3 (two or three barriers)**

Barrier 1: ____________ Strategies used: _____________________________________

Barrier 2: ____________ Strategies used: _____________________________________

Barrier 3: ____________ Strategies used: _____________________________________

**Therapist Comments:**

***ENGAGE* Activities List**

Please identify as many possible pleasurable/meaningful activities as you can, and rate them for difficulty (E=Easy, M=Medium, H=Hard).

1)

2)

3)

4)

5)

6)

7)

8)

9)

10)

***ENGAGE* Action Planner**

1. My goal is: _______________________________________________________
2. Ideas for meeting my goal:

Idea 1: __________________________________________________________

Idea 2: __________________________________________________________

Idea 3: __________________________________________________________

1. Choose best idea (most feasible and most rewarding): __________________________________________________________
2. List steps (What will you do? When? For how long? Who is involved? Where will it happen? What do you need?):

- _____________________________________________
- _____________________________________________
- _____________________________________________
- _____________________________________________

Please complete the questions below *after* you have engaged in your activity.

5. How did you do in achieving your goal?

☺ 😐 ☹

6. If you couldn't do your plan, what got in the way? _______________________________

_________________________________________________________________________________

Barriers Rating Scale

*Emotional Dysregulation Barriers* AR Score___/6

When the patient tries to implement an “action plan,” does s/he:

- Become anxious?
- Become overwhelmed by the task?
- Worry about failure?

When the patient is in session, does s/he:

- Cry easily?
- Become anxious when talking about problems or goals?
- Become visibly upset by the idea of implementing an “action plan”?

*Negativity Bias Barriers* NB score: ___/6

When the patient tries to implement an “action plan,” does s/he:

- Become pessimistic about the plan?
- Come up with potential problems that were not discussed in session?
- Evaluate successful implementation negatively?

When in session, does the patient:

- Seem overly focused on negative information?
- Have difficulty considering the potential for a positive outcome?
- Have trouble thinking of ideas to meet goals because s/he believes nothing will work?

*Apathy Leading to Inertia Barriers* CC Score: ___/6

When the patient tries to implement an “action plan,” does s/he:

- Have trouble starting the plan?
- Experience too many weekly distractions to focus on the plan?
- Seem to forget to do the plan?

When in session, does the patient:

- Seem disorganized and have trouble focusing on one thing at a time?
- Become easily distracted?
- Have trouble with structured tasks?

***ENGAGE* Action Planner (Step 2)**

- 1. My goal is: _______________________________________________________
  2. Ideas for meeting my goal:

Idea 1: __________________________________________________________

Idea 2: __________________________________________________________

Idea 3: __________________________________________________________

- 1. Choose best idea (most feasible and most rewarding): __________________________________________________________
  2. Barrier Strategy is: _______________________________________
  3. List steps (What will you do? When? For how long? Who is involved? Where will it happen? What do you need?):
- _____________________________________________
- _____________________________________________
- _____________________________________________
- _____________________________________________

Please complete the questions below *after* you have engaged in your activity.

6. How did you do in achieving your goal?

☺ 😐 ☹

7. If you couldn't do your plan, what got in the way? _______________________________

_________________________________________________________________________________

**laner**

***ENGAGE* Action Planner (Step 3)**

1. My goal is: _______________________________________________________
2. Ideas for meeting my goal:

Idea 1: __________________________________________________________

Idea 2: __________________________________________________________

Idea 3: __________________________________________________________

1. Choose best idea (most feasible and most rewarding):

__________________________________________________________

1. Barrier Strategy is: _______________________________________
2. List steps (What will you do? When? For how long? Who is involved? Where will it happen? What do you need?):

- _____________________________________________
- _____________________________________________
- _____________________________________________
- _____________________________________________

Please complete the questions below *after* you have engaged in your activity.

6. How did you do in achieving your goal?

☺ 😐 ☹

7. If you couldn't do your plan, what got in the way? _______________________________

_________________________________________________________________________________

**Argument Worksheet**

**Playing the Devil’s Advocate**

Write your reasons for not doing your “action plan” here:

________________________________________________________________________

________________________________________________________________________

Write down why your reasons are not good reasons here:

________________________________________________________________________

________________________________________________________________________

**Getting Your Arguments in Order**

List three good arguments you can use to help you do your “action plan”:

(1)

(2)

(3)

Look at these arguments whenever you start to consider not doing your “action plan.”

**Weighing the Evidence**

**Changing Perspectives**

- 1. **How can I view the situation from a different perspective?**
  2. **How might other family members or friends have thought about or reacted to a similar situation?**
  3. **Think of someone optimistic whose opinion you highly value. How would s/he perceive the situation?**

| Activity | Predicted likelihood of bad outcome (%) | Past experience | Actual experience |
| --- | --- | --- | --- |
|  |  |  |  |
|  |  |  |  |
|  |  |  |  |
|  |  |  |  |
|  |  |  |  |

**Cheat Sheet for Emotion Management**

1. Set aside 10 minutes every day.
2. Find a quite spot where you will not be bothered.
3. Record what your tension level is.
4. Close your eyes and take five slow and deep breaths.
5. Start your practice.
6. End your practice.
7. Record your tension level.

**My practice plan is:**

**Relaxation ○ Imagine ○ Meditation ○ Prayer ○**

| Tension | Monday | Tuesday | Wednesday | Thursday | Friday | Saturday | Sunday |
| --- | --- | --- | --- | --- | --- | --- | --- |
| Before |  |  |  |  |  |  |  |
| After |  |  |  |  |  |  |  |

| Tension | Monday | Tuesday | Wednesday | Thursday | Friday | Saturday | Sunday |
| --- | --- | --- | --- | --- | --- | --- | --- |
| Before |  |  |  |  |  |  |  |
| After |  |  |  |  |  |  |  |

**RELAXATION TRAINING**

Close your eyes and think of a beautiful picture that makes you relax.

Take a deep breath through your nose, like filling your stomach with air.

Hold your breath and count to 5.

Exhale slowly until all air is out (sometimes as you exhale, it may be helpful to whisper a word slowly, for example “relax”).

Wait for 15 seconds.

Repeat.

Please practice these exercises according to the therapist’s recommendations before you apply them in an anxiety provoking situation.

Write down your thoughts and feelings and rate the effectiveness of the exercises on a scale of 1-10 (1=not effective; 10=most effective) after each training session.

Please be aware that there may be an increase in anxiety in the beginning of the training sessions before the exercises are effective.

SLEEP HYGIENE

1. **Do not go to bed unless you are sleepy.** If you are not sleepy at bedtime, then do something else. Read a book, listen to soft music, or browse through a magazine. Find something relaxing, but not stimulating, to take your mind off of worries about sleep. This will relax your body and distract your mind.
2. **If you are not asleep after 20 minutes, then get out of the bed.** Find something else to do that will make you feel relaxed. If you can, do this in another room. Your bedroom should be where you go to sleep. It is not a place to go when you are bored. Once you feel sleepy again, go back to bed.
3. **Begin rituals that help you relax each night before bed.** This can include such things as a warm bath, light snack or a few minutes of reading.
4. **Get up at the same time every morning.** Do this even on weekends and holidays.
5. **Get a full night’s sleep on a regular basis.** Get enough sleep so that you feel well-rested nearly every day.
6. **Avoid taking naps if you can.** If you must take a nap, try to keep it short (less than one hour). Do not take a nap after 3 p.m.
7. **Keep a regular schedule.** Regular times for meals, medications, chores, and other activities help keep the inner body clock running smoothly.
8. **Do not read, write, eat, watch TV, talk on the phone, or play cards in bed.**
9. **Do not have any caffeine after lunch.**

**10. Do not have a beer, wine, or any other alcohol drink before bedtime.**

**11. Do not have a cigarette or any other source of nicotine before bedtime.**

**12. Do not go to bed hungry, but don’t eat a big meal near before bedtime.**

**13. Avoid any exercise within six hours of your bedtime.** You should exercise on a regular basis, but do it earlier in the day (talk to your doctor before you begin an exercise program).

**14. Avoid sleeping pills, or use them cautiously.** Most doctors do not prescribe sleeping pills for periods of more than three weeks. Do not drink alcohol while taking sleeping pills.

**15. Try to get rid of or deal with things that make you worry.** If you are unable to do this, then find a time during the day to get all of your worries out of your system. Your bed is a place to rest, not a place to worry.

**16. Make your bedroom quiet, dark, and a little bit cool.** An easy way to remember this: It should remind you of a cave. While this may not sound romantic, it seems to work for bats. Bats are champion sleepers; they get about 16 hours of sleep each day. Maybe it’s because they sleep in dark, cool caves.

# ***Problem Solving Therapy for Late Life Depression***


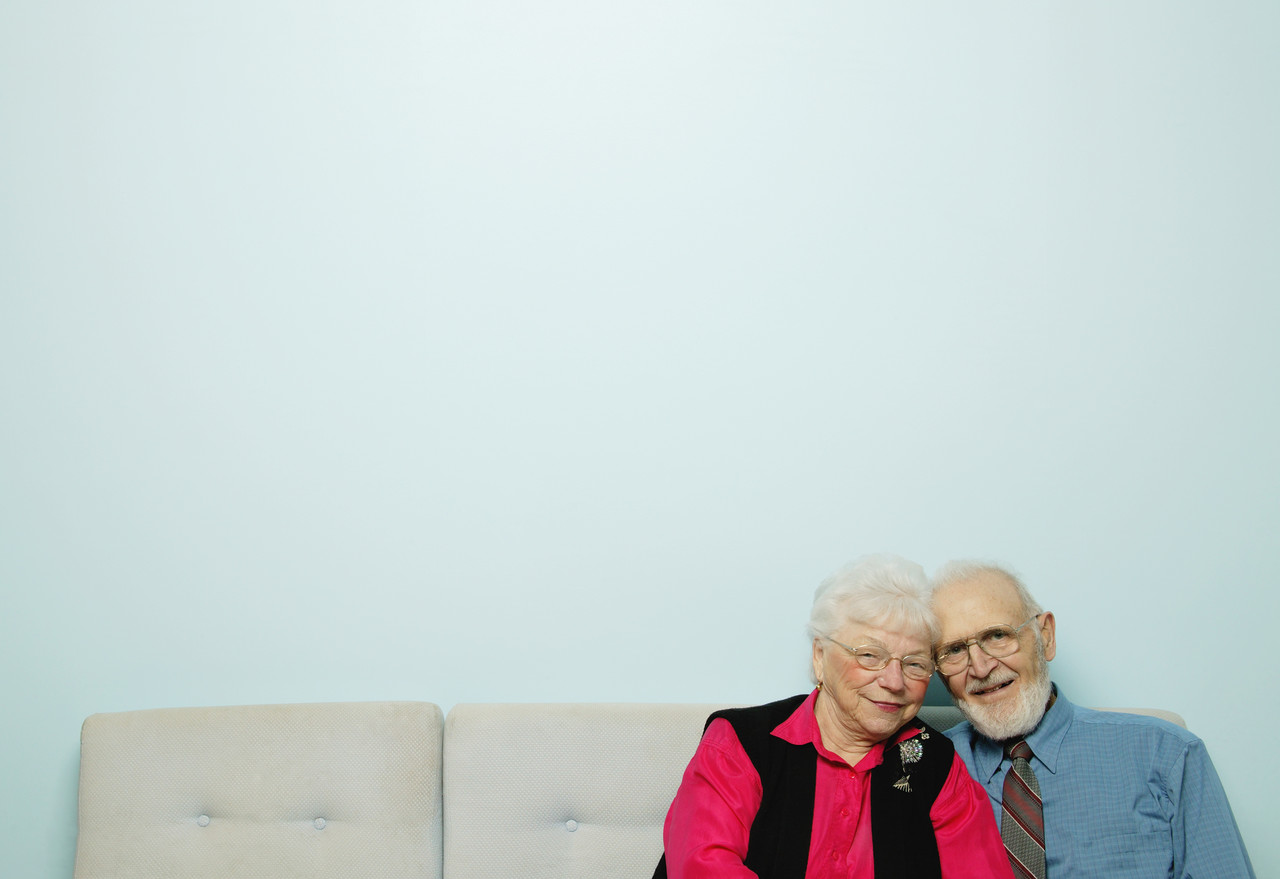


# Table of Contents

# **Chapter 1: Problem Solving Overview page 3**

Chapter 2: Problem Solving Therapy page 5

Chapter 3: The Role of Activity Scheduling in PST

page 17

Chapter 4: Community Resources page 18

Chapter 5: Special Considerations when Working with Older People page 19

Appendix A: Problem List

page 25

Appendix B: Problem Solving Form

page 27

**Chapter 1**

Problem Solving Therapy for Depressed Older Adults

Problem Solving Therapy for Older Adults (PST-OA) is a modification of the Social Problem Solving Therapy manual developed by Arthur Nezu and Thomas D’Zurilla and the Problem Solving Therapy Manual for Primary Care developed for the IMPACT trial. It is important for anyone using this manual to read and refer to two books on Social Problem Solving Therapy: Social Problem Solving Therapy: Theory, Research and Practice (Nezu, Nezu, and Perri, 1989) and Problem-Solving Therapy: A social competence approach to clinical intervention (D’Zurilla and Nezu, 1999). Over the past few years, we have made a number of modifications to PST to make it more user friendly to older adults. These modifications were informed by our experience in using PST, as well as from feedback from our past patients.

The first modification is that the examples given to illustrate the problem solving stages have been modified to focus primarily on issues specific to late life. Our experience has shown that a mix of geriatric and depression specific problems makes the intervention more acceptable to older people. The second modification has been the length of treatment; this version of PST is 9 sessions long. The last modification includes the use of strategies for those with mild cognitive impairments. These modifications are discussed in more detail in Chapter 4.

In order for PST-OA to be effective, one must ensure that the patient: (1) understands the rationale behind the model and (2) understands its application. Educating and socializing the patient to therapy is often important with older persons. Some have never been in therapy before and, therefore, an explanation of how the treatment will work, how often you will meet, and the importance of homework will need to be discussed in the first session. A good geriatric therapist always asks patients what their expectations are of therapy and tries to allay any concerns or misconceptions. Some older people have had psychotherapy in the past, but most likely will not have been exposed to a structured therapy like PST-OA. It is important, in this case, that the patient understand the difference between PST-OA and traditional psychotherapy. Telling the patient that they will be learning a new set of skills rather than relying exclusively on discussion of problems is a key difference between PST-OA and traditional therapies.

PST consists of seven stages that efficiently address psychosocial problems. These stages are: (1) selecting and defining the problem, (2) establishing realistic and achievable goals, (3) generating alternative solutions (4) implementing decision making guidelines, (5) evaluating and choosing solutions, (6), implementing the preferred solution, and (7) evaluating the outcome. Therapists can use these stages in creating a link between their patients and social programs, and in doing so, demonstrate the PST process to their patients with the intent of patients using the process to solve non-case management problems. Therapists and patients work together, using the same logic model to solve both social and psychological problems.

This manual describes how to teach problem solving skills to older adults with MDD. The manual is the result of 15 years of research at UCSF and Cornell on the implementation of PST to older adults with medical illnesses and mild cognitive impairments in several ethnic groups and socioeconomic settings.

Chapter 2 of the manual describes the problem solving process. In Chapter 3, we provide general information regarding aging resources and organizations that may be used in developing action plans for patients with socioeconomic need. In Chapter 4, we discuss issues related to working with older adults, including the impact of ageism on motivation for change and how disability affects treatment. Also included are the PST form and the Problem List described in Chapter 2.

# Chapter 2

## Problem Solving Therapy

This chapter describes the specific stages of PST and how to use the PST form to solve patient problems.

**The Seven Stages of PST**

1. Selecting and defining the problem 5. Evaluating and choosing solutions

2. Establishing realistic and achievable 6. Implementing the preferred goals solution

3. Generating alternative solutions 7. Evaluating the outcome

4. Implementing decision making 8. Activity Scheduling guidelines

Each stage of PST is represented on the PST Form (Appendix A). This form was created with older adults in mind; it has large font to account for vision problems and big writing spaces to account for graphomotor impairments. Below, we discuss each stage of PST and how to complete the form.

***Stage 1: Selecting and Defining the Problem***

*
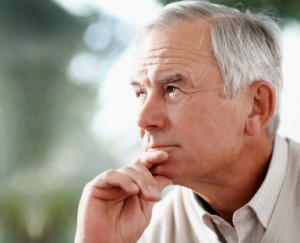
*

The goal for this stage is to succinctly define the selected problem. We feel it is important to note that the most pressing and urgent problems should be selected first, working down the list to the least pressing problems. Patients tend to be too distracted by urgent problems to learn PST. By progressing through problem selection in this manner, patients will be ready to learn the PST process.

Selection of patient problems, however, should progress in the opposite order. Selecting the least complex problems early on in PST facilitates patient understanding of the model, and gives patients the opportunity to achieve success with the model in relatively short order. In our experience, focusing on a sensitive problem can distract the patient from the learning process.

Most patients will describe their problems in vague and unclear terms. It is common to hear patients say that their problem is “procrastination,” “being poor” or “being sick.” While the essence of these terms may be true, the details are missing. What does it mean to procrastinate? Is the patient complaining about paying rent in a timely fashion or attending to a health plan the physician created? How is “being poor” problem for the patient? Is the patient having trouble paying bills or can the patient pay bills but is the patient unable to purchase gifts for friends? As can be seen, these general terms do not help in determining how to solve the problem.

Defining problems involves the following:

- Discussing the specifics of the problem
- Breaking large problems down into small steps
- Using concrete and observable terms to describe the problem

***Discussing specifics of the problem.*** It is practically impossible to solve any problem without a thorough understanding of the problem to be addressed. If the problem is not thoroughly explored prior to developing solutions, then the therapist and patient run the risk of generating inadequate or irrelevant solutions. For patients, this means having them describe in detail the factors that make their situation problematic. Getting information about the unmet needs, why they have not been addressed, and all the barriers and obstacles encountered by patients is important before therapists and patients embark on developing an action plan.

***Breaking down large problems into smaller and more manageable parts.*** Problems are often comprised of a number of smaller, yet distinct, interrelated parts. Failure to differentiate these components leads to an overly vague problem definition that in turn leads to an inefficient action plan. Being poor is a large problem, with many potential issues. One patient complained that being poor affected her ability to several things, such as pay her rent on time, purchase medication, buy new clothing, and purchase gifts for her grandchildren. Each of these areas is a problem in its own right, deserving of its own, separate action plan.

***State the problem in a clear and objective form****.* Once the problem has been broken down into smaller parts and all aspects of the need or problem have been discussed, patients and therapists can define the problem or need succinctly. It is important to describe the problem or need into observable terms, such that patients and therapists will know definitively when the problem or need has been met.

Examples of defined problems versus undefined problems are:

*Undefined* *Defined*

“Poverty” “I cannot pay my electricity bill”

“Paralysis” “I cannot get my mail from the mailbox”

“Procrastination” “I forget to use my walker every day”

Sometimes patients will identify vegetative symptoms of their depression, such as problems with energy, sleep, or motivation, as problems. Although these symptoms are “problematic,” they are not objective life problems and, therefore, are not the best problems to identify for problem solving. Nonetheless, if the patient insists that they wish to address these, or there are no objective life problems that appear to exist, then a symptom may be chosen as the problem area as long as the functional correlates of the symptom are identified. The problem definition is, then, constructed in reference to the functional impairment rather than the symptom. For example, low energy may have the function of decreasing the patient’s ability to do housework. In this case, the problem definition becomes “trouble getting housework done.” Likewise, lack of motivation may interfere with going out of the house to visit with friends, and the problem definition becomes “difficulty doing self care activities.” As patients become more effective in resolving these functional problems, their depression will begin to lift and the symptoms of low energy and motivation will improve.

Once the problem has been succinctly defined, write it down on line 1 of the PST Form.

# *Stage 2: Establishing Realistic and Achievable Goals for Problem Resolution*


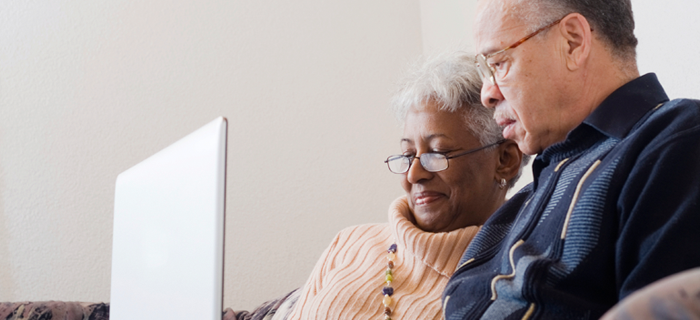


Once the need or problem is defined, the next step is determining what patients would like to see changed. This goal should be clearly and succinctly defined. The goal should also be achievable with a reasonable amount of effort and time. It is important to take into account the balance between the available resources and the time frame for its achievement. Patients and therapists will have long-term goals that they intend to reach by the end of the 9 weeks. These long-term goals can usually be broken down into steps that can be accomplished from meeting to meeting. For instance, one patient had the long-term goal of paying off his debts. This goal was further divided into setting a budget, paying off his utility bills first (before winter), then his credit card bills, and finally his medical bills. These were further divided into smaller, achievable steps, such as finding out the financial programs for which he was eligible. By breaking the goal into smaller goals that could be reached each week, the patient felt a greater sense of success than he would have had his only goal been to relive his debts.

Setting clear behavioral goals is important during Stage 7 when the success of the solution implementation is evaluated. When the set goal stipulates a specific outcome (e.g., getting transportation, calling one’s family) and those outcomes occur, then it is clear that the problem-solving plan worked. Once the goal has been defined, this is recorded on line 2 of the PST Form.

***Stage 3: Generating Multiple Solution Alternatives: Brainstorming***


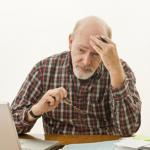


Once the goal has been set, you are now ready to generate a range of potential solutions. Research has shown that depressed people often have a difficulty generating solutions, partly because they are discounting the effectiveness of solutions before adequately defining them. Teaching individuals to creatively think of a range of possible solutions is based on the premise that the availability of a number of alternative actions will increase the chances of eventually identifying particularly effective solutions. In other words, the first idea that comes to mind is not always the best idea. Therefore, it should be emphasized to the patient that they should try to generate as many solutions as possible via "brainstorming" techniques. Successful brainstorming involves:

- Listing at least five solutions
- Solutions must address the goal
- Patients and therapists must withhold judgment
- For patient problems, the solutions must come from the patient, not therapist

***List at least five solutions.*** The number of solutions generated is important. The greater the number of potential solutions, the greater the chances for successful resolution of the problem or need. Having a large number of solutions also allows patients and therapists to combine ideas when it is practical to do so.

Therapists using this approach may find it difficult to generate more than one or two obvious solutions for a particular need. For instance, if a patient needs to get to medical appointments and is not linked to Para transit, the obvious solution for the therapist is to link the patient to that service. However, for the patient’s benefit, it is worthwhile to consider other options so that the patient can observe that there is usually more than one way to solve a problem, and the patient can observe the therapist employ the decision making strategies described later.

***Solutions must address the goal.*** Sometimes, patients tend to list whatever idea comes to mind without considering the relevance to the problem. This is a relatively rare, but it does happen. If, when generating solutions, patients begin to list what appear to be irrelevant solutions, therapists should ask how the solution is related to the goal, **e**ven before the brainstorming process ends. Sometimes, patients’ solutions may seem irrelevant because there is another aspect to the problem that was not discussed. In this case, problems should be redefined and goals newly specified.

***Withhold judgment until the next stage.*** Do not judge the ideas until the brainstorming process is completed, otherwise a potentially successful and novel solution may be prematurely abandoned. Evaluation of the feasibility of each option is left for the subsequent stage (Stage 4: Implementing Decision-Making Guidelines: Pros and Cons).

The solutions should be listed in the grid on item 3 of the PST form.

# *Stage 4: Implementing Decision-Making Guidelines: Pros versus Cons*


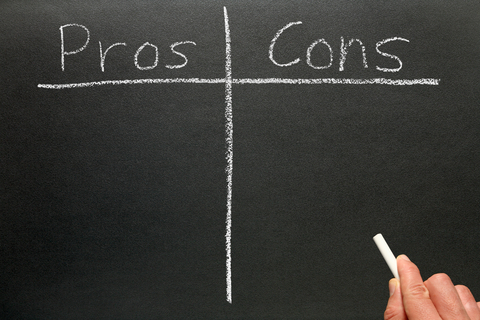


The purpose of stage 4 is to strategically evaluate the alternative solutions by implementing decision-making guidelines. Therapists and patients create a list of the “pros” and “cons” for each potential solution. This involves asking the following questions:

- Does the solution meet long term and short term goals?
- What is the impact the solution will have on the patient, other people and/ or society?
- Is the solution feasible?

***Does the solution meet long term and short term goals?*** The main point of this question is to determine the feasibility of being able to implement the solution between meetings. Some solutions may be excellent at addressing a need in the long run, but if selected, should probably be combined with another solution that can meet a short-term goal so that patients experience some success from week to week.

***What is the impact the solution has on the patient, other people, and/or society?*** The main point in asking this question is to determine if the solution will create some other, unforeseen problem. For instance, relying on family for transportation may meet a goal of going to weekly physical therapy appointments, but may result in overburdening the family. It is not enough to meet the goal; a good solution also minimizes any negative impact on others.

***Is the solution feasible?*** The main point in asking this question is to determine whether or not patients and therapists actually have the resources to enact the solution. A therapist may be able to help patients with transportation by driving them to appointments, but that is unlikely to be a feasible solution.

# **As with all of the problem-solving stages, it is ideal for the patient to derive their own pros and cons list. However, there are two occasions in which it is acceptable for the therapist to introduce information. The first is when the patient is overlooking an extreme negative consequence for themselves or others. This would certainly include a consequence of significant physical or emotional harm to oneself or others, and may include episodes of interpersonal conflict, such as with a spouse or co-worker. The second instance is when the patient mentioned an advantage or disadvantage earlier during the session, such as during the brainstorming phase, but appears to have forgotten this in the current stage. In this case the patient has already demonstrated that they are aware of the issue and the therapist is only reminding them to include it in the decision analysis process. The pros and cons of each solution should be listed in the gird next to each solution under item 3 of the PST form.**

***Stage 5: Evaluating and Choosing the Solution(s)***


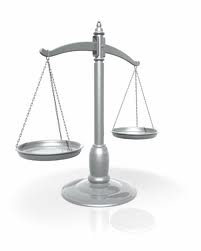


The next stage in problem solving is to prompting the patient to compare the solutions along their pro versus con dimensions. This involves:

- A thorough examination of all solutions
- Comparing solutions

Determining which solution has the fewest cons and most pros.

***A thorough examination of all solutions****.* Therapists should begin this stage with a careful review of the relevant pros and cons for each solution. This within-solution evaluation involves determining if a solution has more against it than for it. This process helps to begin weeding out less effective solutions, and opens up discussion on how a weak solution may be strengthened.

***Comparing solutions.*** After the merits of each solution have been detailed, therapists and patients can compare the solutions to one another. The solution that should be selected is the one with the smallest number of negative consequences and the largest number of positive outcomes associated with it.

Some patients find this stage of problem-solving initially difficult to achieve alone, ruminating about possible solutions without being able to choose one or overlooking important decision-making guidelines established in the previous stage. When patients choose a solution without appropriately reviewing the pros and cons, therapists should point it out and bring the evidence to their attention. Likewise, if a potential solution is left on the drawing board which seems an obvious choice to the therapist based upon the decision analysis, the therapist should assure that a deliberate reasoning process was used in deciding not to include this solution as an option. Awareness of using the evidence to choose the solution should be verified by engaging the patient in a brief discussion and review of the important decision-making information after they have chosen a solution.

The chosen solution should be written out on item 4 of the PST form.

# *Stage 6: Implementing the Preferred Solution(s)*

Once chosen, the steps required to achieve a solution are identified and planned. This is the **action plan**. To create a clear action plan, patients and therapists must:

- List the steps necessary for implementing the solution
- Consider all potential obstacles

***List the steps necessary for implementing the solution****.* Therapists and patients must consider all the steps necessary for implementing a solution. As an example, the steps involved in linking patients to Para transit involve calling Para transit and reviewing eligibility, determining if there is a waiting list, collecting the appropriate forms and dropping the off to the patient, mailing in the forms, and calling to follow-up on the status of the application. Specifying when each task is to take place is also important. Using the same example, therapists may indicate that the first call to Para transit will happen as soon as they return to the office. For patients, determining the timing of steps is very important in supporting adherence to the plan. Patients are more likely to implement an action plan if they start the first step as soon as the therapist leaves the home.

***Consider all potential obstacles****.* Patients must identify and choose tasks that they feel comfortable implementing. Therapists should assure that the tasks are sufficient to satisfy the requirements of the solution as well. Sometimes this means that the solution may need to be broken down into more simple sub-steps. In its extreme form, this may mean going back to the original problem definition and beginning the process again. More often it requires returning to the decision-making guidelines and re-evaluating the solutions. A new solution may be chosen if the original solution requires an action the patient feels unable to carry out.

This stage is sometimes rushed due to time constraints, as it is the last stage completed during the visit. Therapists should be aware that the action steps are the culmination of all the good work that has preceded it. Therefore, to rush through this stage is to lose the value obtained from having completed the previous stages. The successful outcome of the entire PST process rests upon its proper completion. It is well worth the few extra minutes to do this stage well and ensure a successful outcome for the patient. The action plan is filled out in item 5 of the PST form.

*Stage 7: Evaluating the Outcome*

The final stage is actually completed at the start of the subsequent meeting. Patients and therapists should have completed or attempted to complete the action plans set in the previous session, and should have recorded the outcome of these tasks on item 6 of the PST Form. In addition to determining if a solution met a goal, proper evaluation of outcomes includes answering the following questions:

- Were you satisfied with the outcome?
- Did you learn anything new about the problem?
- Is there anything you would have done differently?

***Were you satisfied with the outcome?*** The review of homework should be followed by asking patients about their sense of satisfaction with their effort and the impact of their success on their mood. Particularly during early treatment sessions, patients may state that the success had no impact on their mood. On these occasions therapists should review the PST model and emphasize that they certainly are no worse off for having solved a problem. It is important to encourage persistence. When mood improvement is reported, therapists should point out the link between effective problem solving and achieving a positive mood state.

***Did you learn anything new about the problem?*** This question is particularly pertinent to reviewing solutions that do not succeed in meeting needs or goals. In discussing patient failures, therapists should always communicate that they see patients’ potential for effective coping, and thus facilitate a positive problem-solving orientation. This is also an opportunity to indicate that the problem-solving process is useful in failed situations too, and that failures often result in more information about the problem that was not available earlier. The therapist may point out that solutions usually do not work out because not all the facts were available when attempting to solve the problem; because no one ever truly has all the facts when initially solving a problem, failures are part of the process, are part of life, and are opportunities to improve our ability to cope with the problem.

The final task to be accomplished in Stage 7 is to link the patient’s efforts to the PST model and reinforce their understanding of the rationale for the intervention. If patients continue to be motivated participants in treatment and continue to apply the problem solving strategy when treatment has ended, they must understand and endorse the value of the approach. An opportunity to make the case for the rationale for PST is when patients have been successful and report being satisfied with their efforts or when their mood has improved. Before moving on to choosing another problem for the current session, the therapist must make an effort to assure that the patient understands the connection between problem solving efforts and a positive mood state.

***Chapter 3***

***The Role of Activity Scheduling in PST***


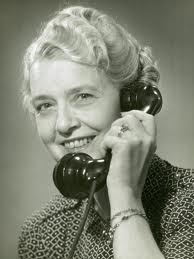


Activity Scheduling is a strategy for helping patients incorporate pleasant and satisfying activities into their lives. Activity Scheduling is based on the research of Peter Lewinsohn, a psychologist who showed that depressed individuals engage in significantly fewer pleasurable events than do non-depressed individuals. Lewinsohn’s theory of depression assumed that the lack of pleasant events is an important contributor to depression and that when people are depressed they are less likely to seek out pleasant events. Thus, a downward spiral is established in which lack of pleasant events promotes depression, which in turn leads to fewer pleasant events, thereby worsening depression.

It is important to note that PST is a more difficult skill to acquire than it appears to be. It is important for therapists to watch closely for signs that a patient is having difficulties with a particular stage of the model. In those situations, it may be necessary for the therapist to spend time reviewing exactly what to do at that particular stage. For this reason, we have developed supplemental reading materials and exercises for the therapist to use in these situations.

## Chapter 4

**Community Resources**

Social services and resources will vary from county to county. Further, availability of resources is often dependent on the economic and political environment. It is important for therapists to remain updated on social services changes. This can be done through regular contact with the Area Agency on Aging, contact with the division of geriatric mental health in the department of public health, and through county publications that are updated yearly and list all available resources and social services for older adults.

Suggested resources for therapists are:

- American Association for Retired Persons (AARP)
- National Coalition on Aging (NCOA)
- Area Agency on Aging (AAA)
- Departments of Health and Mental Health
- Adult Protective Services

Membership in local aging organizations is also important, and we suggest strongly that therapists become members. In San Francisco, the main organization for providers for elderly people is the Coalition of Agencies Serving the Elderly (CASE). In Westchester County, the main organization is the Westchester Department of Senior programs and Services (WDSPS).

The San Francisco Family Service Agency and the WCDSPS sponsor educational programs on managing older populations. While largely educational in nature, this is another good place to meet other providers and network with agencies.

At your training, we will provide you with the most recent county resource list for aging programs. It is your responsibility to keep this list updated.

## Chapter 5

## Special considerations when working with older people

# *Ageism and boundaries*

In American society, aging is seen as a time of despair, disability, and pain. One need only look to the cosmetics industry to see the plethora of products aimed at reducing the appearance or suggestion of age – hair dies, Rogaine, Botox, wrinkle creams – all products suggesting that the natural course of aging is something to avoid. Ageism is a negativistic belief about older people that is present in people of all age groups. Because most of us grew up with some ideas about older adults, and because our society is rife with stereotypes about aging, our attitudes toward age and aging need to be considered when working with older adults in the context of psychotherapeutic interventions.

An older patient’s attitudes toward a therapist can take many forms. Indeed, some have noted that because elders have more years of experience, he or she can have feelings about the working relationship that stem from experiences in any life stage, including the family of origin, nuclear and extended family, and other relationships. A therapist must be aware of this range of possibilities, and of the fact that a patient can see the therapist as a parent, a spouse, a child or grandchild, or an expert.

For a therapist, ageism will likely arise in work with older adult patients. For many therapists, a lack of professional training with older adults leaves them especially vulnerable to their own fears about aging. Often, these feelings stem from his/her positive or negative stereotypes about older people and his/her own fears regarding infirmity and aging. For example, one younger therapist observed that she had difficulty interrupting or confronting her elderly patients while conducting PST. She connected this to being taught as a child to be extremely respectful to her grandparents and other elderly people. Once she had identified the source of her behavior, she could more accurately assess the situations that required her to be more assertive with her patients.

Relevant to ageism are the decisions made by therapists regarding boundaries in the working relationship. For good reasons, many mental health professionals have been trained to maintain an emotional distance and not accept gifts or make physical contact with patients. However, it is true that among the current cohort of older adults, it may be relatively common to offer a small gift or a hug to a therapist, and to become insulted if rejected. This may be especially true for older adults from certain cultures. For example, one therapist provided in-home therapy to an elderly Filipino couple, who insisted on serving tea and a snack during each session. The therapist did her research and learned that this behavior was typical of this culture and cohort; despite her initial discomfort with the ritual, she assessed the impact on the working relationship to be minimal and decided to allow the tea to continue. The main lesson here is to perform a truly comprehensive assessment when conceptualizing the elderly patient and making decisions about boundaries, taking into account personal, cohort-based, and cultural factors. The following questions may be useful in evaluating ageist attitudes and boundaries within the working relationship with an elderly patient. Several of these were inspired by the “contextual, cohort-based, maturity, specific-challenge” (CCMSC) model of case conceptualization developed by Knight for use with older adults.

*Questions to consider about the patient*:

**What cohort does this patient come from? How might that affect interactions with me? How does it affect the patient’s coping strategies?**

**What is this person’s cultural background?**

What is this patient’s specific family history**,** independent of age or cohort?

What does aging mean to this person?

# **What is this person’s current social world, and how does it fit with his/her current needs?**

*Questions therapists can ask themselves*:

# **What does “aging” and “old age” mean to me?**

# **What are my stereotypes about older people?**

How comfortable am I with my own aging and loss of function, and that of my loved ones?

What is my cohort and how does that affect my behavior and my perspective?

What did I learn as a child about older people, and about how to interact with them?

How I do want to be seen by this older person?

***Adapting PST for Older Adults***

Despite the overall similarities between working with older and younger adults, experienced geriatric therapists agree that some adaptations may be needed to make the treatment maximally effective. These adaptations include taking time to socialize older adults to the process of PST, adjusting the pace of PST to account for age-related changes in information processing, and allowing flexibility in the delivery of PST to overcome medical and physical barriers to care. At the same time, older adults bring unique strengths to therapy that therapists can capitalize on such as past experience and wisdom.

***Accounting for Changes in Information Processing***

Although research shows that older people maintain a significant degree of mental flexibility and can learn new tasks, older adults do learn somewhat differently than younger persons. There are a number of cognitive changes associated with aging that should be attended to when providing psychotherapy to an older patient. The most relevant changes to psychotherapy are those associated with cognitive slowing, decreased fluid intelligence, and working memory. Taken together, these changes indicate that psychotherapy – which relies on the ability to draw inferences, process new material, and recall information – often must be delivered at a slower pace, and in a multifaceted way.

***Cognitive slowing.*** The speed to which we react to stimuli, and hence process information, slows considerably as we age. Although slowed reaction time does not necessarily interfere with the ability to process new and/or abstract material, new information should be presented more slowly and over a longer period of time to counteract the effects of cognitive slowing.

Psychotherapies adapted for older populations tend to be structured so that new material raised in treatment is reviewed a number of times and presented through a number of modalities. In PST, this process involves first giving older patients a rationale for a new skill and elucidating the relevance to their problems. Next, the therapist demonstrates the new skill with a generic example, and finally engages the patient in the skill using a patient example. In following this process, the therapist can check to make sure that the older patient understands the application of the new skill and can successfully practice the skill between sessions.

**Decreased fluid intelligence.** While the overall reasoning ability of older adults is not impaired, and they have a vast store of previous learning (“crystallized intelligence”), the rate at which they can process new information and make inferences (“fluid intelligence”) is slowed. Although the details of memory functioning among older adults are quite complex, there is consensus that working memory, an aspect of memory functioning that is responsible for processing information prior to long-term memory formation, becomes less efficient with age. Again, providing repeated exposure to new information is important to help ensure adequate learning. Another technique, which makes use of intact crystallized intelligence to improve information processing in psychotherapy, is to rely on patients’ vast stores of previous experiences. Life review, a technique commonly found in reminiscence therapies, is an excellent tool for linking new material to older patients’ past experiences. This technique is best illustrated in a case example below.

## Case Example: Life Review in PST

Mr. J. was a disabled 80-year-old man referred to problem-solving therapy for major depression. During the course of therapy, Mr. J. learned the steps involved in PST through the “say-it, show-it, do-it” method, but he was still struggling with understanding the process of PST and was not applying the model between sessions. More out of frustration than therapeutic gain, the therapist decided to spend a session letting Mr. J talk about his problems in a free-form fashion. As Mr. J. spoke about his depression, he began discussing the job he had before he became disabled and how good it made him feel to be the “go-to” person for the roadblocks faced by his company when rolling out a new product. As the therapist listened to Mr. J. talk about how he managed to solve problems in one particularly complex situation, the therapist noticed similarities between Mr. J.’s problem solving process at work and the PST model. She then asked, “Is that how you usually solved problems at work? Did you typically follow those steps?” Mr. J. discussed a few more examples of how he solved problems at work, and as he spoke, the therapist tracked the terms he used for his problem-solving steps and used the PST worksheet to record the process Mr. J. took to solve these problems. After a few instances of life review, the therapist showed Mr. J. what she had done and drew a parallel between his work style and PST. Mr. J. thought for a moment, began nodding his head, and then, as if a light bulb had gone off, he said: “Has this been what you’ve been trying to get me to do? Well, why didn’t you say so?” Using the patient’s life review material, the therapist was able to successfully teach PST skills to Mr. J. and subsequently help him overcome his depression.

Although this example describes use of life review in a relatively unstructured manner, therapists may conduct more targeted life review around specific issues. For example, it can be helpful to talk about previous times when the patient faced similar issues and how they managed to resolve or cope with those issues earlier in their life. Ideally, such a review can remind patients of coping skills they already have. Even if patients did not cope effectively with those issues in the past, however, life review discussions can still serve as a valuable learning tool in therapy.

***Contextual adaptation.*** As stated previously, for some older adults a number of practical and health-related barriers may exist, requiring certain contextual modifications to the therapy. The most common therapy adaptations to address contextual issues include: a) relaxing the therapeutic frame to accommodate fatigue, illness, and psychosocial demands and b) adapting psychotherapy elements to address common physical disabilities and coordinate with other care providers.

***The therapeutic frame.*** The traditional therapeutic frame can be a barrier to the delivery of psychotherapy in older populations. The typical expectations that patients come in weekly for appointments, that treatment be delivered during a 50-minute time span, and that it occur in specialty mental health settings may be hard for many older patients to meet. The therapeutic frame must remain flexible with regard to treatment location, session length, and access. Because many older adults are coping with caregiving crises, temporary disability due to short-term illnesses or the exacerbation of chronic illnesses, or ongoing medical illnesses that require a number of appointments, being able to participate in regular psychotherapy can be a complicated goal to attain. To account for these factors, therapeutic approaches that allow for flexibility in treatment have been developed for late-life psychotherapy. These include modifying psychotherapy for non-mental health settings, briefer sessions, and using the telephone and/or written materials.

***Accounting for physical disabilities.*** Disabilities common in frail elderly (e.g., impairments in vision, hearing, or mobility) also can impede the progress of therapy when no adaptations are undertaken. Ideally, the therapist assesses disabilities and attempts to facilitate patients’ receipt of needed medical and social services (e.g., medical treatment, getting new glasses or dentures). The therapeutic process also may benefit from close, ongoing collaboration with other health care professionals, particularly in working with frail elderly with multiple medical problems and medications.

For patients with reading impairments (due to vision loss or illiteracy), audio-taping sessions for at-home review can be used to reinforce session information. For some patients, treatment forms should be modified with larger print and with larger writing spaces to accommodate changes in fine motor skill (e.g., due to arthritis or stroke).

For patients with hearing loss, the therapist must be aware of the degree of impairment. In some cases, sitting closer to older patients and near the ear that is less affected by hearing loss can greatly help with communication. Speaking slowly and in low tones (particularly important for female therapists) can also help the older patient hear the material. If these methods prove ineffective, microphones connected to headphones the patient wears can be used to amplify therapists’ voices. Relying on written communication is another option that has been used successfully in some cases.

Finally, chronic or acute physical impairments may interfere with patients’ ability to attend and sit through sessions. Therapy sessions may need to be briefer due to fatigue or pain. Therapists also need to assess for and attempt to correct any environmental barriers for these patients, such as lack of transportation, lack of wheelchair accessibility, loose rugs, or poor lighting.

In summary, psychotherapy for older adults typically means more sessions to process information. It also mean that new information, whether it is in the form of learning skills or exploration of experience, may need to be reviewed with the older adult to ensure comprehension and assimilation of information. Because of illness and other competing demands on time and energy, the therapeutic frame must be flexible, but not to the detriment of the patient. Finally, adaptations to address physical impairments may be important to consider.

Strengths of older adults

In spite of the challenges that may arise in psychotherapy with older adults, older adulthood also can be a time of growth, and older adults often retain strengths that therapists can use to maximize therapeutic benefit of the work together. For example, although some cognitive functions may be less efficient, research suggests that, compared to younger adults, older adults “have a larger repertoire of experience from which to operate, use more effective strategies, and better integrate emotional information.” These findings suggest that it can be beneficial for therapists and their older patients to explore the strengths they have developed over their lifetime and ways to use those strengths to approach current issues. Even “mistakes” or regrets can be used to determine different courses of action for the future. Elders in distress are likely to overlook or minimize their assets and past accomplishments, and the therapist may need to be very proactive in assessing and identifying patient strengths during the session. For example, one elderly woman described herself as not accomplishing much in life, then went on to talk about raising three children on her own after her husband’s early death. She minimized her role in raising her children, although further discussion revealed that she had worked very hard and frequently expressed love and support to her children. Such discussions can serve to build elders’ sense of self-worth and encourage them in dealing with current issues.

Another potential strength of older adults is the presence of more complex emotionality (i.e., multiple emotions in response to an event or issue) to explore and integrate in therapy. This ability can be beneficial in psychotherapy, especially when working on the complex and multi-faceted issues common to old age.

##### **Appendix A**

##### **Problem List**

**PST Problem List**

Family Home

1. _______________________________ 1.___________________

2. _______________________________ 2. ___________________

3. _______________________________ 3. ____________________

Financial

1. _________________________________

2. _________________________________

3. _________________________________

Social

1. __________________________________

2. __________________________________

3. __________________________________

Health

1. _________________________________

2. _________________________________

3. _________________________________

Appendix B

**Problem Solving Form**


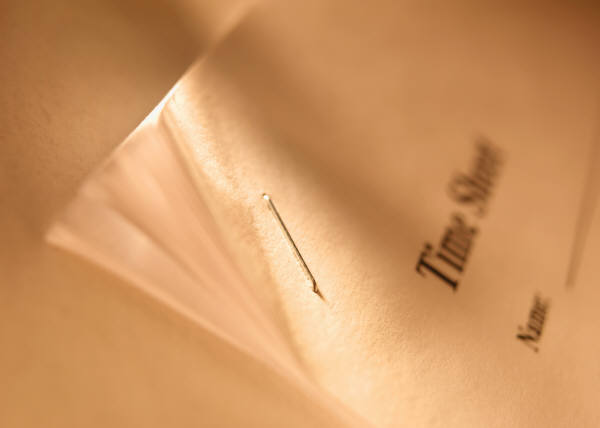


**Problem Solving Worksheet**

Date:_______________ Name:________________ Visit:______

Review of Progress:_________________________________________

1. Problem Definition:_______________________________________

2. Goals:_________________________________________________

3. Solutions:

4. Pros Cons

|  |  |  |
| --- | --- | --- |
|  |  |  |
|  |  |  |
|  |  |  |
|  |  |  |

5. Choice:________________________________________________

6. Steps:

a)

b)

c)

d)

7. Satisfaction? ☹ 😐 ☺

0 1 2 3 4 5 6 7 8 9 10

8. Activities for week:___________________________________
